# GTM-3, an Extra-Large Pore Enantioselective Chiral Zeolitic Catalyst

**DOI:** 10.1021/jacs.2c01874

**Published:** 2022-05-03

**Authors:** Ramón de la Serna, David Nieto, Raquel Sainz, Beatriz Bernardo-Maestro, Álvaro Mayoral, Carlos Márquez-Álvarez, Joaquín Pérez-Pariente, Luis Gómez-Hortigüela

**Affiliations:** † Instituto de Catálisis y Petroleoquímica, ICP-CSIC. C/ Marie Curie 2, Madrid 28049, Spain; ‡ Instituto de Nanociencia y Materiales de Aragón (INMA-CSIC), Universidad de Zaragoza, Zaragoza 50009, Spain; § Laboratorio de Microscopias Avanzadas (LMA), Universidad de Zaragoza, Zaragoza 50018, Spain

## Abstract

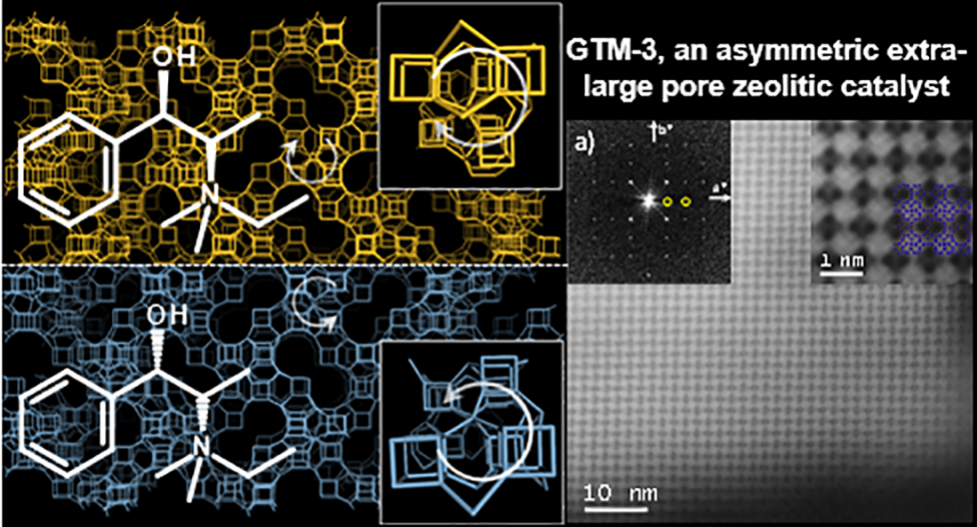

The development of chiral zeolitic catalysts possessing extra-large pores and endowed with the capability of enantioselectively processing bulky products represents one of the greatest challenges in chemistry. Here, we report the discovery of GTM-3, an enantio-enriched extra-large pore chiral zeolite material with -ITV framework structure, obtained using a simple enantiopure organic cation derived from the chiral pool, *N*,*N*-ethyl-methyl-pseudoephedrinium, as the chiral-inductor agent. We demonstrate the enantio-enrichment of GTM-3 in one of the two enantiomorphic polymorphs using the two enantiomers of the organic cation. Interestingly, we prove the ability of this zeolitic material to perform enantioselective catalytic operations with very large substrates, here exemplified by the catalytic epoxide aperture of the bulky *trans*-stilbene oxide with alcohols, yielding unprecedented product enantiomeric excesses up to 30%. Our discovery opens the way for the use of accessible chiral zeolitic materials for the catalytic asymmetric synthesis of chiral pharmaceutical compounds.

## Introduction

Chirality, the property of an object of not being superimposable to its mirror image, is crucial in living organisms: since its most remote origin, life decided to work asymmetrically using only certain enantiomers of the biochemical building units: l-aminoacids for proteins and d-sugars for nucleic acids. A critical consequence emerged from such asymmetry: the metabolism of all organisms differentiates between the two enantiomers of a chiral compound.^1^ This was sadly evidenced by the drug thalidomide, which was administered as a racemic mixture to pregnant women, with one of the enantiomers provoking teratogenic effects on newborns;^2^ it was later evidenced that thalidomide racemized under physiological conditions, so even if single enantiomers would have been administered, teratogenic effects would have still occurred.^3^ In any case, such tragedy evidenced the importance of developing methods to separate or asymmetrically synthesize the different enantiomers of chiral compounds.^4^

In this context, zeolites have been proposed as ideal candidates to achieve active catalysts able to perform enantioselective operations.^5−11^ Zeolites are crystalline microporous materials based on periodic silicate frameworks having regular pores and cavities of molecular dimensions.^12,13^ Confinement within the void space of microporous frameworks prompts the size/shape discrimination between guest species having small steric (geometric) differences,^14^ triggering the characteristic shape-selectivity of zeolite catalysts. Chirality is also a geometric property that is manifested in the asymmetric three-dimensional (3D) structure of a molecule, and so confinement in microporous spaces within zeolites can be exploited to discriminate between the mirror-image molecular structures of enantiomers, as long as the microporous framework is chiral. To date, more than 250 different zeolite frameworks have been discovered,^15^ but only a few of them are chiral,^6,16−20^ usually containing helicoidal channels.

The main strategy used to promote the crystallization of chiral zeolites has been through the use of chiral organic cations as structure-directing agents (SDA);^21−24^ these organic entities control the porosity of the nascent zeolite framework through a *template* effect, where they transfer their geometric properties (size and shape) to the void volume of the zeolite framework through the development of host–guest nonbonding interactions.^25^ This geometric host–guest relationship can also be extended to exploit the asymmetric nature of chiral SDAs and the potential transfer of such chirality into an asymmetric void space of a zeolite, and in turn, into a chiral zeolite framework whose handedness would reflect that of the SDA. However, for such a transfer of chirality to occur, a true *template* effect, in the sense of a close geometrical relationship established between SDAs and zeolite frameworks, must occur during crystallization. This is not often the case, and although many chiral organic cations have been used over the years,^5,26^ only very recently this strategy has been, for the very first time, proved successful for producing an enantio-enriched chiral zeolite.^7,27,28^ Davis and co-workers prepared a rationally designed organic cation where the asymmetric nature of the SDA was transferred to the STW chiral framework, resulting in enantio-enriched materials. Interestingly, the authors found enantioselective properties of their material both in adsorption and catalytic processes.

**Scheme 1:**
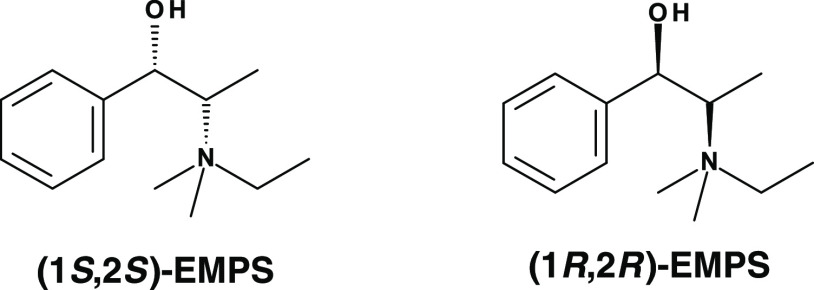
Molecular Structure of the Two Enantiomers of *N,N*-Ethyl-methyl-pseudoephedrinium

Considering the potential application of chiral zeolites as actual serviceable catalysts for the pharmaceutical industry, three crucial factors must be addressed: (i) the chirality of the zeolite framework must be transferred to the asymmetric catalytic process; (ii) zeolites should have extra-large pores (>0.7 nm) to enable processing large substrates typically used as drug precursors; and (iii) chiral SDAs used for their synthesis should be accessible at a reasonable cost. Ideally, the SDA should be prepared from organic species available from the chiral pool, where nature readily provides enantiopure chiral precursors derived from natural products. In this line, we have systematically been exploring the use of alkaloids (1*R*,2*S*)-ephedrine and (1*S*,2*S*)-pseudoephedrine (Supporting Figure S1) as chiral blocks for building SDAs.^29−34^ In the course of these investigations, we have recently discovered the crystallization of GTM-3 (GTM stands for *Grupo de Tamices Moleculares*), a germanosilicate zeolite material with the -ITV framework, using (1*S*,2*S*)-*N*,*N*-ethyl-methyl-pseudoephedrinium as SDA (hereafter referred to as EMPS) (Scheme 1).^35^ -ITV, which was originally discovered by Corma and co-workers as ITQ-37,^16^ is one of the most interesting zeolite frameworks because of its extremely high porosity and, especially, of its chiral nature comprising a gyroidal channel system with 30-ring windows. The synthesis of the original ITQ-37 used a complex achiral polycyclic organic dication (Supporting Figure S2-A), thus resulting in a racemic crystalline conglomerate. Later on, three other polycyclic cations were reported to also direct the crystallization of the -ITV framework (Supporting Figure S2-B,C,D).^36−38^

## Results and Discussion

GTM-3 zeolite was prepared by the hydrothermal method from gels with molar composition 0.25EMPS:0.75SiO_2_:0.25GeO_2_:0.25HF:6.5H_2_O, which were crystallized for 6 days at 100 °C; worth noting is the low temperature and low concentration of the SDA used (0.25), making the synthesis more cost-effective. X-ray diffraction (XRD) patterns (Supporting Figure S3) showed that GTM-3 has the -ITV chiral framework originally reported by Corma et al.^16^ Importantly, the synthesis of enantiopure (1*S*,2*S*)- or (1*R*,2*R*)-EMPS cations was straightforward, starting from the corresponding commercially available chiral alkaloid precursor, (1*S*,2*S*)-pseudoephedrine or (1*R*,2*R*)-pseudoephedrine; hence, GTM-3 could be prepared from the two EMPS enantiomers, which is crucial to have a proper control over the chiral properties (Supporting Figure S4).

GTM-3 only crystallized at temperatures below 110 °C; higher temperatures led to amorphous materials, probably as a consequence of the thermal decomposition of the organic cation. On the other hand, GTM-3 did crystallize at temperatures as low as 60 °C (Supporting Figure S5), though at a slower rate, evidencing the high efficiency of our SDA. Such low crystallization temperatures, which are uncommon in zeolite synthesis, are critical since enantiomeric energy differences in host–guest SDA/zeolite diastereomeric pairs during crystallization are expected to be low and could potentially be disrupted by excessively high thermal energies. Si/Ge ratios in the gel from 3 to 5 allowed the crystallization of GTM-3, resulting in materials with Si/Ge ratios around 2.6–2.8 (as determined by EDX) and crystal size in the range of 80–130 nm (Supporting Figure S6).

The strong structure-directing ability of EMPS toward the -ITV framework is supported by the fact that related cations with very similar molecular structures like methyl-((1*S*,2*S*)-*N*,*N*-dimethyl-pseudoephedrinium) or propyl-((1*S*,2*S*)-*N*,*N*-methyl-propyl-pseudoephedrinium) analogues, or even the (1*R*,2*S*)-diastereoisomer derived from (1*R*,2*S*)-ephedrine ((1*R*,2*S*)-*N*,*N*-ethyl-methyl-ephedrinium) (Supporting Figure S7), did not allow the formation of this framework (at least under the conditions tested here). This suggests a strong host–guest geometrical match between EMPS and the -ITV framework. Surprisingly, EMPS has a molecular structure very different from that of the SDAs reported so far to direct the crystallization of -ITV, having a much smaller size, with only one ring (in contrast to previous SDAs that all contained four rings; see Supporting Figure S2) and with high flexibility. The occlusion and integral incorporation of EMPS within GTM-3 was confirmed by thermogravimetric analysis, ^13^C CP MAS NMR, and CHN elemental analysis (Supporting Figures S8 and S9 and Table S1).

GTM-3 was stable upon removal of the organic content to generate permanent porosity in the absence of humidity, as typical for germanosilicates (Supporting Figures S10 and S11). N_2_ adsorption/desorption isotherm evidenced the high porosity of GTM-3 (Supporting Figure S12), with a micropore volume of 0.31 cm^3^/g and an external surface area (BET) of 866 m^2^/g, similar to those of the original ITQ-37.^16^

**Figure 1:**
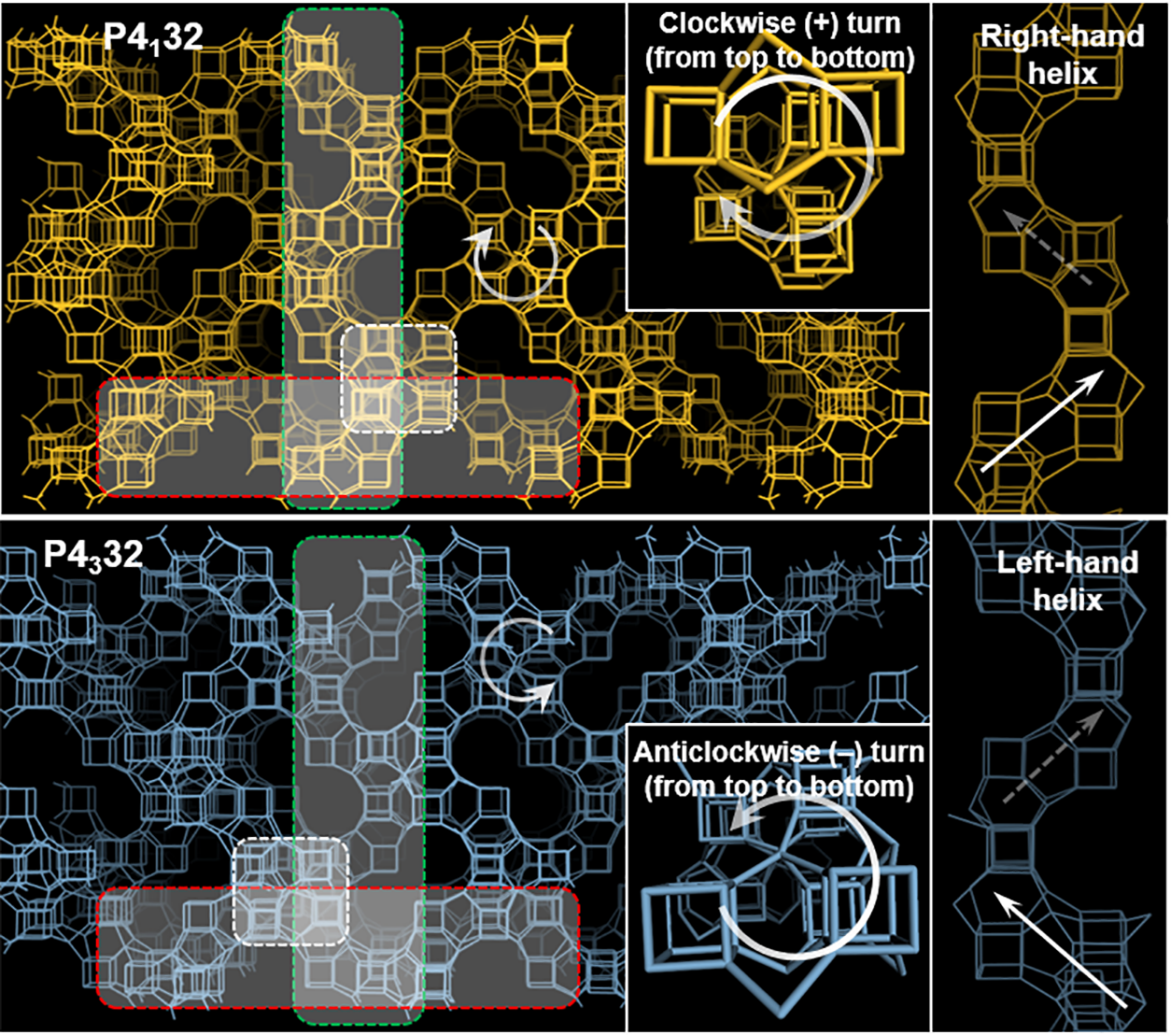
Chiral -ITV framework with *P*4_1_32 (top) or *P*4_3_32 (bottom) enantiomorphic space groups, showing the helicoidal chain units (in the three directions, like spiral staircases, dashed rectangles) with right-hand and left-hand helices, respectively, turning 90° in clockwise (+90°) or anticlockwise (−90°) directions (viewed from top to bottom).

The main interest of GTM-3 is that it comprises a chiral framework (-ITV) that can crystallize in two enantiomorphic polymorphs, *P*4_1_32 or *P*4_3_32, with helicoidal spiral staircase-like chain units of opposite handedness (Figure 1), and hence with potential asymmetric catalytic activity. The chiral enantiopurity of the SDA, (*S*,*S*)- or (*R*,*R*)-EMPS, prompts a potential transfer of chirality from the SDA to the -ITV framework, especially considering the strong host–guest -ITV/EMPS match. Of course, for a transfer of chirality from the organic cations to occur, a prerequisite is the retention of their enantiopurity during the hydrothermal treatment. To assess this, the organic material occluded within GTM-3 was extracted by dissolving the zeolite in HF (the integrity of the released EMPS was confirmed by ^13^C NMR, Supporting Figure S9), and the resulting solution was analyzed by polarimetry. Specific rotations of +68° (for GTM-3 obtained in the presence of *S*,*S*-EMPS) and −58° (for GTM-3 obtained with *R*,*R*-EMPS) were obtained. These values were similar in magnitude and with the same sign as those of the original *S*,*S*-EMPS and *R*,*R*-EMPS solutions (+66° and −64°, respectively), unambiguously demonstrating the retention of enantiopurity of EMPS cations after occlusion within GTM-3.

**Figure 2:**
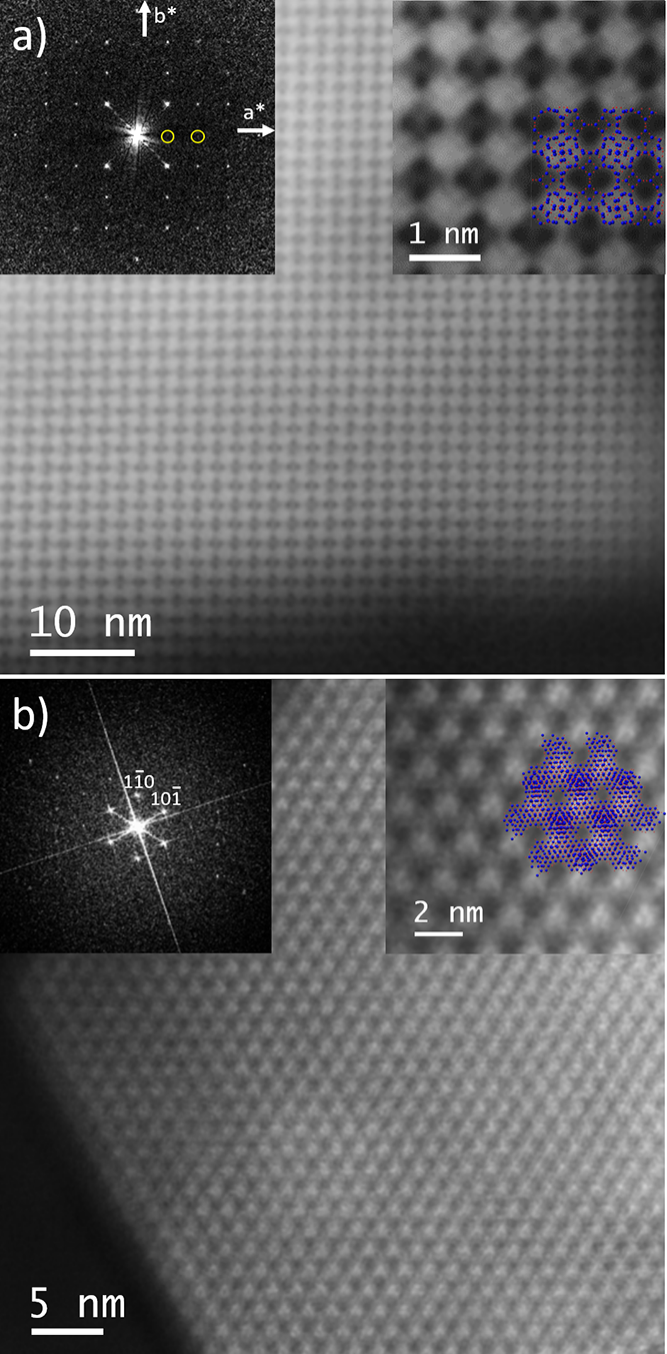
Cs-corrected STEM-ADF analysis: (a) along the [001] projection with the FD inset (a magnified image is presented on the top right corner with the model superimposed); (b) [111] projection with the FD and a closer observation, with the model superimposed (Si in blue and O in red) shown in the inset.

The next step was to determine if a transfer of chirality from the SDA to the framework, in the form of an enrichment (or even exclusive crystallization) in *P*4_1_32 or *P*4_3_32 -ITV polymorphs occurred when using each of the two enantiomers of EMPS. The most direct way to analyze such enantio-enrichment would be to solve the absolute configuration of many individual GTM-3 crystals by single-crystal X-ray diffraction, which was not possible because of the very small (nanometric) size of GTM-3 crystals. Alternatively, HRTEM methods have been recently proposed to solve the absolute configuration of STW chiral zeolite crystals.^27,39^ High-resolution spherical aberration-corrected (Cs-corrected) scanning transmission electron microscopy (STEM) coupled with an annular dark-field (ADF) detector was employed to confirm the framework topology proposed, study the crystallinity of the materials, and provide additional information on the chirality of GTM-3. One aspect that was previously described by Corma and co-workers was the low stability of this zeolite,^16^ probably associated with its low framework density, preventing high-resolution imaging. By working in STEM mode and controlling the electron dose not higher than 800 e^-^/Å^2^, it was possible to visualize the GTM-3 framework. Figure 2a displays the high-resolution observation along the [001] orientation, where the largest rings are observed. The Fourier diffractogram (FD) shown in the inset can be indexed as either *P*4_1_32 or *P*4_3_32 space groups, where the systematic absences fulfilling the reflection conditions *h*00: *h* = 4*n* are evidenced (marked by yellow circles) along a* and b* axes. A magnified visualization of the structure with the model superimposed is also depicted in the inset. Unfortunately, even at such a low electron dose, obtaining different orientations of the same crystal that would shed light on the absolute configuration of the zeolite enantiomer present was not accessible due to the low stability, which resulted in disintegration while it was being tilted toward the other zone axis. A similar analysis was performed on a different crystal, which was tilted along the [111] orientation (Figure 2b), where the FD indexed assuming *P*4_1_32 or *P*4_3_32 is also shown in the inset. A zoomed visualization displays a perfect correlation with the structural model, as shown in the top right inset.

**Figure 3:**
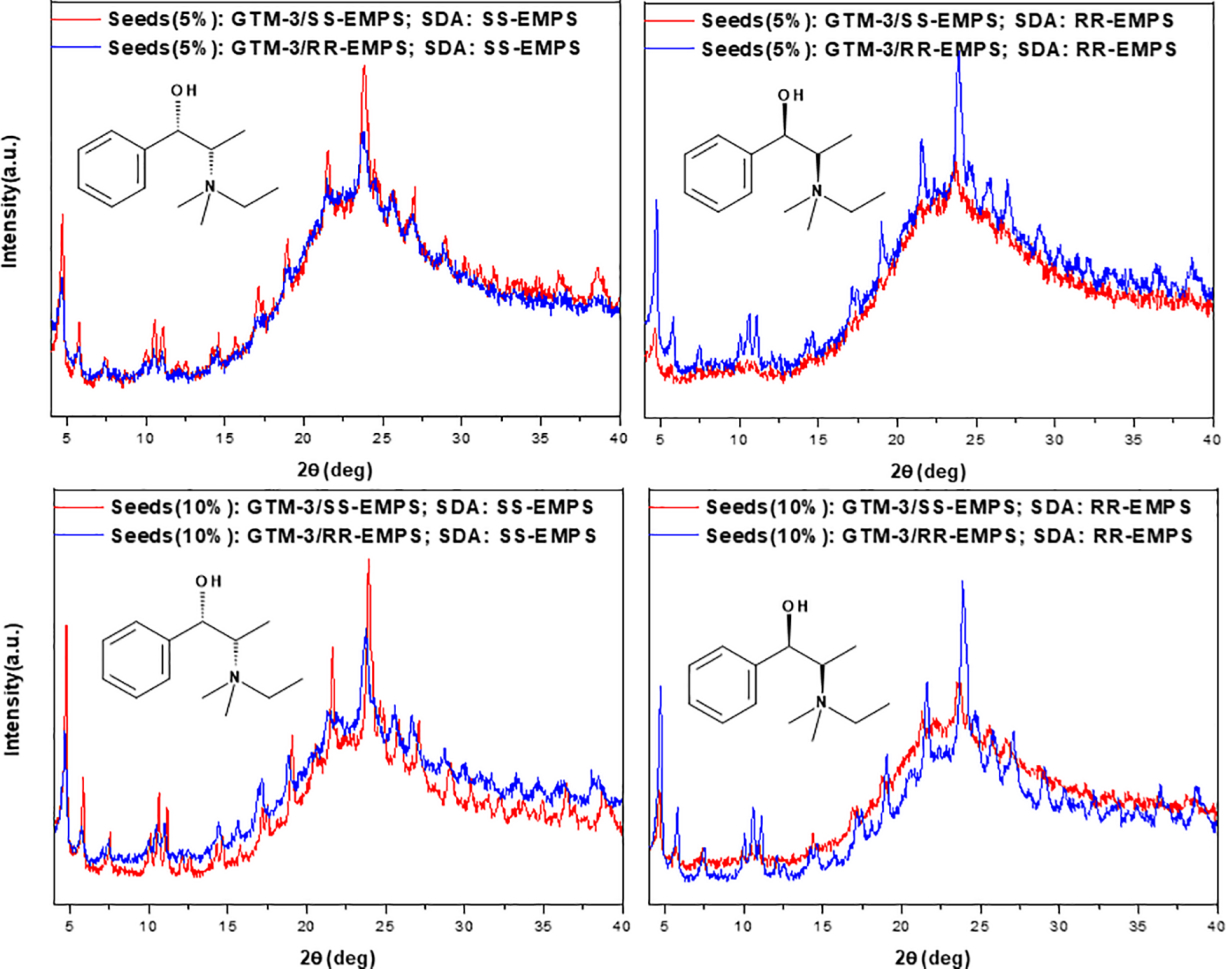
Enantioselective crystallization: XRD patterns of samples prepared with *SS*-EMPS (left) or *RR*-EMPS (right) as SDA in the gel, using 5% (top) or 10% (bottom) of GTM-3 seeds previously prepared with *SS*-EMPS (red lines) or *RR*-EMPS (blue lines). Crystallization was carried out at 100 °C for 72 h.

Therefore, we devised an indirect strategy to study the potential enantio-enrichment of GTM-3. First, we analyzed the effect of adding GTM-3 seeds to the synthesis gels; to clearly recognize a seeding effect, we partially hindered the spontaneous crystallization of GTM-3 by decreasing the Ge content (Si/Ge = 8), which will make the formation of the zeolite more difficult (Ge is known to stabilize this framework). As expected, the presence of seeds notably improved the crystallization of GTM-3 (Supporting Figure S13). Once the role of seeds was confirmed, GTM-3 materials were obtained with either (*S*,*S*)-EMPS (GTM-3/*SS*-EMPS) or (*R*,*R*)-EMPS (GTM-3/*RR*-EMPS) to be used separately as seeds. Then, new synthesis gels with (*S*,*S*)-EMPS as SDA were prepared and seeds obtained with the same enantiomer (GTM-3/*SS*-EMPS) or the opposite enantiomer (GTM-3/*RR*-EMPS) were added in two separate experiments to determine whether the secondary growth of GTM-3 assisted with seeds recognized the chirality of the original seeds. These experiments were repeated using (*R*,*R*)-EMPS as SDA in the secondary gel, again with the two types of seeds. Interestingly, results showed that when the SDA enantiomer added to the gel was the same as the one used for the preparation of the seeds, a higher crystallization rate was clearly observed (Figure 3), evidencing an enantioselective crystallization of GTM-3 in the presence of seeds. As expected, experiments with (*S*,*S*) and (*R*,*R*) SDAs showed a mirror-image behavior. Such enantioselective crystallization occurred both with 5% (top) or 10% (bottom) of GTM-3 seeds (see Supporting Figure S14 for additional experiments). These results evidence that the -ITV/EMPS systems in the secondary growth are able to differentiate the chirality of the original seeds, and in turn that GTM-3 is not a racemic conglomerate but displays an intrinsic chirality in the form of an enrichment in one of the enantiomorphic polymorphs (*P*4_1_32 or *P*4_3_32). However, because of the low stability of GTM-3 under the electron beam, at present, we cannot discern if GTM-3 zeolite is enantiopure (only one polymorph crystallized) or enantio-enriched in one enantiomorphic polymorph.

The next and definitive proof of evidence for the enantio-enrichment of GTM-3 comes from its enantioselective behavior when used as a catalyst. A typical asymmetric catalytic reaction is the ring aperture of epoxides with nucleophiles catalyzed by acids,^40^ which is significant for practical use since it leads to synthetically important β-alkoxy/amino-alcohols (using alcohols or amines as nucleophiles, respectively), which constitute versatile intermediates for the synthesis of biologically active products.^41^ Previous simulation studies had shown the difficulty of the -ITV framework in recognizing different enantiomers of organic sorbates due to the large size of the chiral pores;^9^ hence, selection of a proper reaction substrate was not trivial. With the aid of molecular simulations, we selected the aperture of *trans*-stilbene oxide because of its size and the potential ability of -ITV to recognize the chirality of the two substrate enantiomers; simulations suggested a stronger enantio-discrimination ability of -ITV for *trans*-stilbene oxide compared to that of styrene, butane, or octane epoxides (Supporting Figure S15, Tables S2 and S3). To test the activity of our catalysts for bulky products, 1-hexanol was used as the nucleophile agent (see details in Supporting Figures S16 and S17). Since Ge-containing zeolites have been shown to display catalytic activity in reactions catalyzed by weak Lewis acid sites associated with tetrahedrally coordinated Ge atoms embedded in the framework,^42−45^ Al-free GTM-3 materials were first tried as catalysts. However, as Al-containing samples with tetrahedral framework Al have also been successfully synthesized (Supporting Figure S18), providing a potential source of Brønsted acidity after calcination, Al-containing samples have also been tested in the reaction.

**Figure 4:**
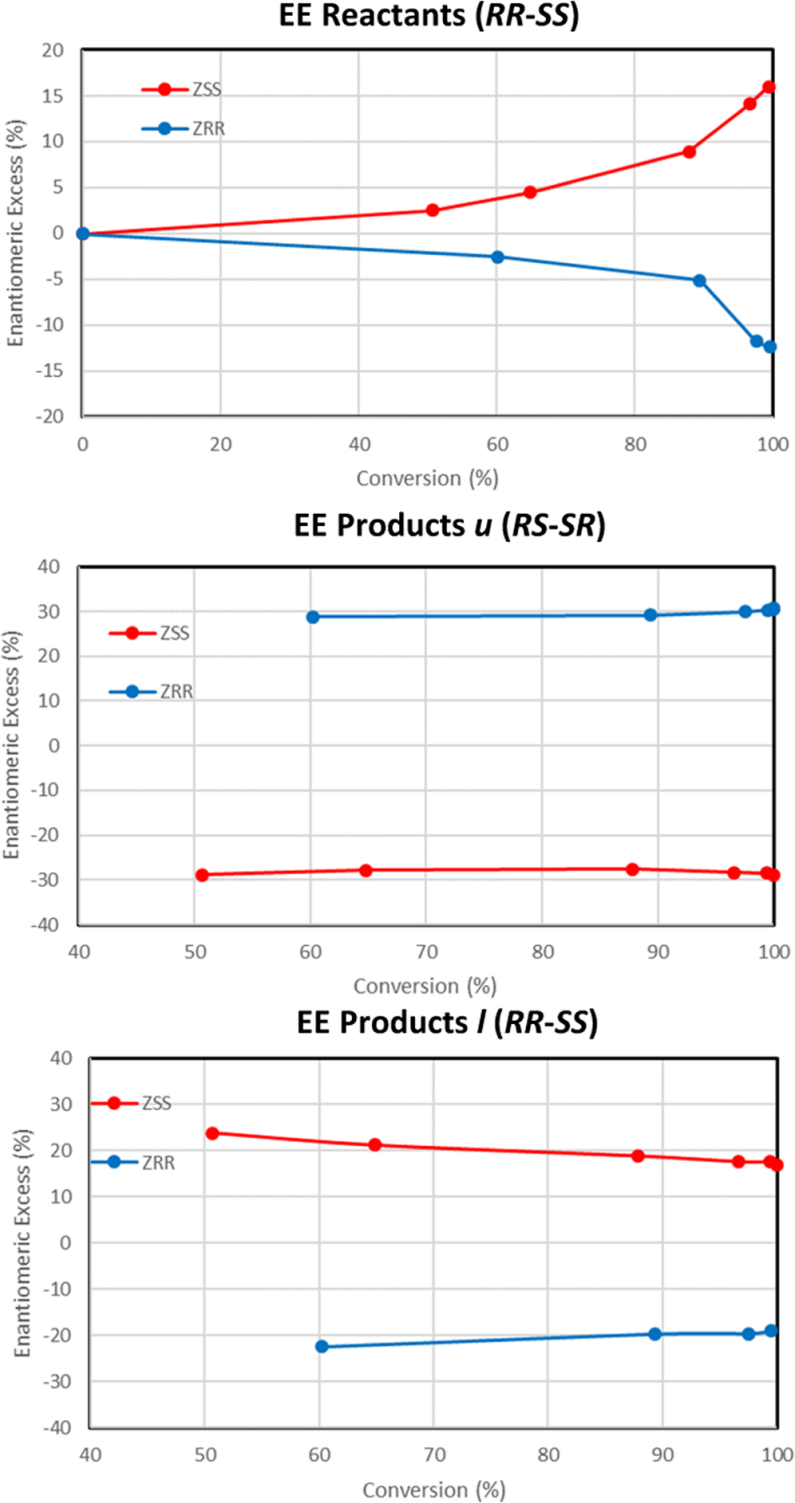
Enantiomeric excesses (%) of reactants (top), *u*-products (middle), and *l*-products (bottom) as a function of conversion, using Al-free GTM-3 catalysts obtained with *SS*-EMPS (red) or *RR*-EMPS (blue), for the *trans*-stilbene epoxide aperture with 1-hexanol.

**Scheme 2:**
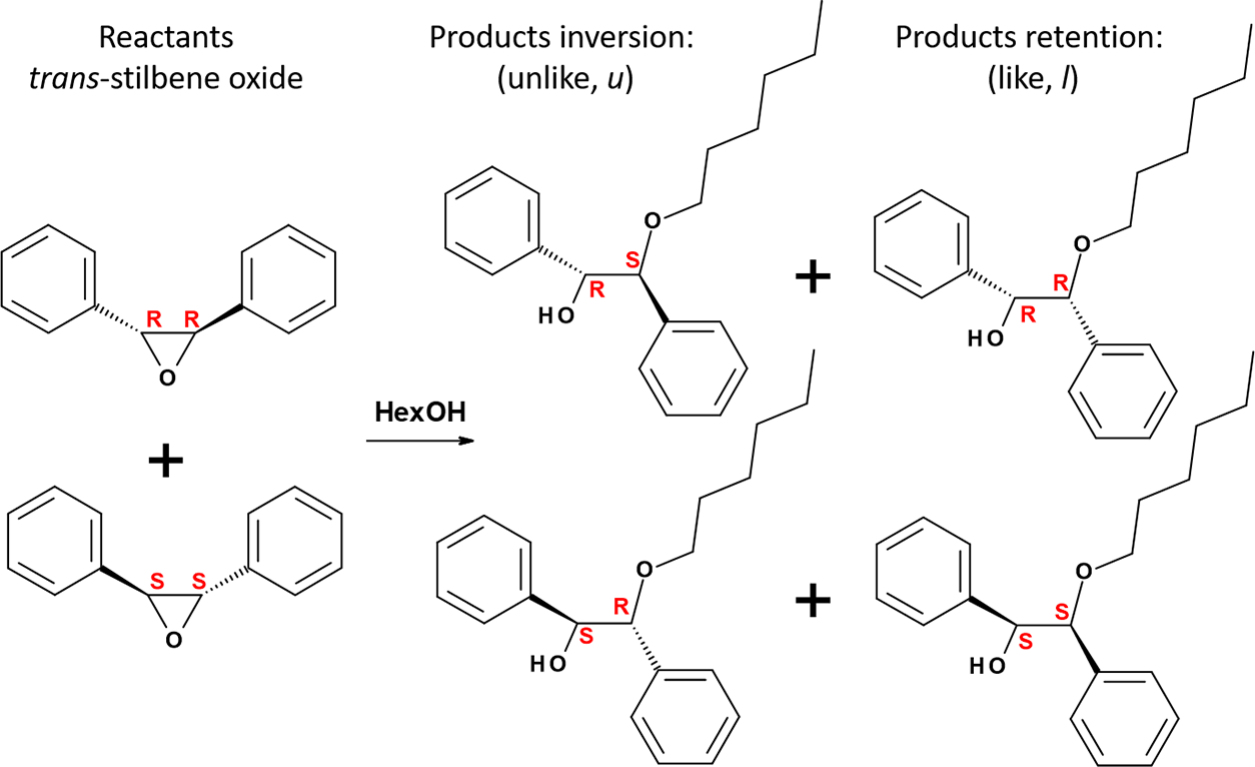
Ring Aperture of Chiral *trans*-Stilbene Oxide with 1-Hexanol Giving Inversion *u*-Products (*R*,*S* + *S*,*R*) or Retention *l*-Products (*R*,*R* + *S*,*S*). Species in Columns Are Enantiomers

Since the epoxide substrate is chiral (having two enantiomers with *R*,*R* or *S*,*S* configuration), the reaction can give two types of chiral products, with inversion (unlike, ‘*u*’, *R*,*S* or *S*,*R*) or retention (like, ‘*l*’, *R*,*R* or *S*,*S*) configurations (Scheme 2). Interestingly, when using Al-free GTM-3 catalysts, relatively high enantiomeric excesses (*ee*) up to ∼30% were observed for inversion *u*-products (*RS*/*SR*) (Table 1 and Figure 4 middle), which remained constant as conversion increased. The reaction proceeded very fast, with complete conversions in 30 minutes. As should be, opposite *ee*’s were obtained when using *SS*- or *RR*-GTM-3 zeolites. Such *ee* values involved that our catalyst produced almost twice (∼1.9) of one enantiomer than the other, which constitutes a substantial difference. Slightly smaller *ee*’s of ∼23% were found for retention *l*-products (*RR*/*SS*) (Table 1), which, in this case, slightly decayed with increasing conversion (Figure 4 bottom). Moreover, the enantiomeric excess of reactants increases with conversion (Figure 4 top), evidencing that one enantiomer reacts faster than the other. Significantly, the *ee*’s obtained with our catalyst (up to 30%) are higher than those previously reported for the other enantioselective STW (ca. 10–23%)^27^ or β enriched in polymorph A (∼5%)^7^ chiral zeolites available. GTM-3 catalysts could be recycled, keeping the same conversion and *ee* (Supporting Figure S19). As expected, no activity at all was found for as-made SDA-containing GTM-3 materials (nor in the absence of catalyst), evidencing that enantioselectivity comes from the inner pores of the chiral framework. On the other hand, ^19^F MAS NMR (Supporting Figure S20) showed a signal at −9 ppm, which could be assigned to F in D4Rs with Ge pairs (−7.5 ppm in STW) or Ge close clusters (−10.5 ppm),^46^ or possibly to a mixture of them, indicating that the active sites of GTM-3 are associated with such Ge pairs and/or clusters. Indeed, pyridine adsorption–desorption experiments monitored by FTIR spectroscopy (Supporting Figure S21) showed the presence of both Lewis and Brønsted acid sites of weak strength (pyridine was nearly fully desorbed at 300 °C from Lewis sites and 200 °C from Brønsted sites), the latter probably induced by dissociative adsorption of water molecules on Ge,^44^ as well as other acid sites of higher strength characteristic of this material (they have not been reported for other germanosilicates),^42,44,45^ where pyridine was retained even at 300 °C.

**Table 1:** Enantiomeric Excesses Obtained for the Different Chiral Products with GTM-3 Catalysts at Medium and High Conversions

		enantiomeric excess (*ee*) (%)	
zeolite	conversion (%)	inversion (*u*): *RS*/*SR*	retention (*l*): *RR*/*SS*
*SS*-GTM-3	50.7	–28.8	+23.8
	99.4	–28.5	+17.5
*RR*-GTM-3	60.2	+28.8	–22.3
	99.5	+30.3	–19.0

Very similar results were observed for Al-containing GTM-3 catalysts (Supporting Figure S22), though with a slightly higher proportion of secondary products; as expected, *ee*’s close to zero were obtained when Al-GTM-3 materials were prepared using a racemic mixture of *SS*- and *RR*-EMPS as SDA. Hence, it seems likely that the enantioselective catalytic activity of GTM-3 is provided by the acidity associated with tetrahedral Ge pairs or close clusters, which could eventually be assisted by the presence of silanol (SiOH) and germanol (GeOH) structural groups. In this context, it is worth noting that the selectivity toward *u*- and *l*-products, giving similar values of ∼40% and ca. 35–40%, respectively, with *u*/*l* ratios of 1–1.1, strongly differs from that of the same reaction carried out with homogeneous sulfuric acid catalysis, where the ratio was around 9 in favor of *u*-products (Supporting Figure S16).

We finally studied the effect of the size of the reactants (and transition states) over the enantioselective activity of GTM-3. Interestingly, if we shrink the size of the alcohol nucleophile from 1-hexanol to ethanol, the *ee*’s decrease from 30 to ∼10% of the *u*- products (Supporting Figure S23). Moreover, if styrene oxide is used as the epoxide (instead of *trans*-stilbene oxide) (Supporting Figure S15), no enantioselectivity is found either with ethanol or 1-hexanol as nucleophiles, in line with our molecular simulations (Supporting Table S3). This evidences that for the chirality of GTM-3 to manifest, a proper chiral host–guest size match must occur: guest species have to be large enough to interact with a suitable portion of the walls and feel the helicoidal nature of the micropores, as was found for the STW framework,^7,27^ providing experimental evidence to previous theoretical statements.^9^

## Conclusions

The discovery of GTM-3 resolves one of the most challenging quests in materials science by combining the very large pore size of its framework with the enantioselective discrimination ability in asymmetric catalytic processes of industrial interest. It is worth noting that this type of epoxide ring-aperture reaction represents a highly valuable tool in medicinal and organic chemistry since important β-amino-alcohols, used for the synthesis of β-blockers, insecticidal agents, chiral auxiliaries or oxazolines, and β-alkoxy-alcohols, precursors of α-alkoxy ketones, α-alkoxy acids, and enol ethers, and a range of bioactive natural and synthetic products, are obtained through this route. Very importantly, the use of accessible chiral precursors available from the chiral pool will surely facilitate its potential use in the pharmaceutical industry due to the associated cost-efficiency. Interestingly, both enantiomers of the chiral precursor are available, and hence both enantiomorphic zeolites can be readily prepared. Work is currently underway to further improve the enantioselective properties of these materials in other catalytic processes of industrial interest. On the other hand, heteroatoms like Al, Ti, or Sn could potentially be introduced in GTM-3 framework positions to induce new catalytic sites, in particular for asymmetric redox chemistry, of fundamental relevance in the pharmaceutical industry. Moreover, the extra-large pores combined with the presence of silanol groups in ordered framework positions open the way for anchoring different catalytic functionalities through grafting, and even for the formation of metal nanoparticles/clusters within confined chiral spaces, broadly opening the scope of chiral potential applications of GTM-3.

## Supporting Information Available

Additional experimental details, methods, characterization, molecular schemes, and reaction data (PDF)
