# Supplementary material for: GTM-3, an Extra-Large Pore Enantioselective Chiral Zeolitic Catalyst
Source: J Am Chem Soc. 2022 May 3;144(18):8249–56. doi: 10.1021/jacs.2c01874 (PMC9100664; doi:10.1021/jacs.2c01874)
Supplement: Supplementary file 1 — ja2c01874_si_001.pdf [file ja2c01874_si_001.pdf]

GTM-3, an extra-large pore enantioselective chiral zeolitic catalyst

Ramón de la Serna,[a] David Nieto,[a] Raquel Sainz,[a] Beatriz Bernardo-Maestro,[a] Álvaro Mayoral,[b] Carlos Márquez-Álvarez,[a] Joaquín Pérez-Pariente,[a] and Luis Gómez-Hortigüela[a]*

[a] *Instituto de Catálisis y Petroleoquímica, ICP-CSIC. C/ Marie Curie 2, Madrid 28049, Spain*

[b] *Instituto de Nanociencia y Materiales de Aragón (INMA-CSIC). Universidad de Zaragoza, Zaragoza 50009, Spain. Laboratorio de Microscopias Avanzadas (LMA). Universidad de Zaragoza, Zaragoza, Spain*

Abstract: The development of chiral zeolitic catalysts possessing extra-large pores and endowed with the capability of enantioselectively processing bulky products represents one of the greatest challenges in chemistry. Here we report the discovery of GTM-3, an enantio-enriched extra-large pore chiral zeolite material with -ITV framework structure, obtained by using a simple enantiopure organic cation derived from the chiral pool, N,N-ethyl-methyl-pseudoephedrinium, as the chiral-inductor agent. We demonstrate the enantio-enrichment of GTM-3 in one of the two enantiomorphic polymorphs by using the two enantiomers of the organic cation. Interestingly, we prove the ability of this zeolitic material to perform enantioselective catalytic operations with very large substrates, here exemplified by the catalytic epoxide aperture of the bulky trans-stilbene oxide with alcohols, yielding unprecedented product enantiomeric excesses up to 30 %. Our discovery opens the way for the use of accessible chiral zeolitic materials for the catalytic asymmetric synthesis of chiral pharmaceutical compounds.

Table of Contents

Experimental Procedures A. Details of the synthesis of the SDAs.

Experimental Procedures B. Details of the synthesis of GTM-3.

Experimental Procedures C. Details of the general physico-chemical characterization.

Experimental Procedures D. Details of the polarimetry study.

Experimental Procedures E. Details of the asymmetric catalytic reaction experiments.

Experimental Procedures F. Details of the characterization of acid sites by FTIR spectroscopy analysis of adsorbed pyridine.

Supplementary Fig. S1. Molecular structure of diastereomeric alkaloids (1*R*,2*S*)-ephedrine (left) and (1*S*,2*S*)-pseudoephedrine (right) available from the chiral pool.

Supplementary Fig. S2. SDAs that have been reported to direct the formation of the –ITV framework, compared with (*S*,*S*)-EMPS.

Supplementary Fig. S3. XRD pattern of as-made GTM-3.

Supplementary Fig. S4. XRD patterns of GTM-3 prepared with (1*R*,2*R*)- (top) or (1*S*,2*S*)- (bottom) enantiomers of EMPS.

Supplementary Fig. S5. XRD pattern of GTM-3 prepared at 60 ºC for 14 days.

Supplementary Fig. S6. A. TEM picture of as-made GTM-3. **B,C:** FESEM pictures of as-made GTM-3.

Supplementary Fig. S7. SDAs that directed the formation of the –ITV framework (top, *SS*-EMPS, left, and *RR*-EMPS, right), and related cations that did not (bottom).

Supplementary Fig. S8. A. TGA of GTM-3 in air (solid line), and weight derivative (right, DTG). **B,C,D.** TGA of GTM-3 in He (**B**), coupled to mass spectrometry (**C** and **D**).

Supplementary Tab. S1. CHN elemental analysis of GTM-3.

Supplementary Fig. S9. ^{13}C NMR spectra of EMPS iodide in aqueous solution (D_2O, middle, red), confined within GTM-3 (top, blue), and in aqueous solution after dissolution of GTM-3 (black, bottom).

Supplementary Fig. S10. In-situ XRD patterns of GTM-3 at increasing temperatures.

Supplementary Fig. S11. XRD pattern (performed under inert atmosphere) of calcined GTM-3.

Supplementary Fig. S12. N_2 adsorption/desorption isotherm of calcined GTM-3.

Supplementary Fig. S13. XRD patterns of GTM-3 materials obtained with a Si/Ge ratio of 8 in the gel, in the absence or in the presence of seeds.

Supplementary Fig. S14. Repetition of enantioselective crystallization: XRD patterns of samples prepared with *SS*-EMPS or *RR*-EMPS as SDA in the gel, using 5 % of GTM-3 seeds previously prepared with *SS*-EMPS or *RR*-EMPS.

Supplementary Fig. S15. Molecular simulations for the selection of the epoxide.

Table S2. Molecular dimensions (expressed as averaged molecular shadow lengths during MD simulations) of the different epoxides studied computationally.

Table S3. Averaged interaction energies (in kcal/mol epoxide) of one enantiomer of the epoxide molecule loaded in the two enantiomorphic -ITV frameworks.

Supplementary Fig. S16. NMR characterization of the stilbene oxide aperture with 1-hexanol.

Supplementary Fig. S17. HPLC settings and selected chromatograms.

Supplementary Fig. S18. ^{27}Al MAS NMR of Al-containing GTM-3.

Supplementary Fig. S19. Recycle of GTM-3 catalyst.

Supplementary Fig. S20. ^{19}F MAS NMR of GTM-3.

Supplementary Fig. S21. FTIR spectra recorded after adsorption of pyridine.

Supplementary Fig. S22. Enantiomeric excesses (%) of reactants, *u*-products and *l*-products as a function of conversion, using Al-containing GTM-3 catalysts.

Supplementary Fig. S23. HPLC chromatograms (and enantiomeric excesses (%)) of *u*-products (*RS*/*SR*) for the *trans*-stilbene epoxide aperture with ethanol.

Experimental Procedures

A) Details of the synthesis of the SDAs

Synthesis of (1*S*,2*S*)- and (1*R*,2*R*)-N,N-ethyl-methyl-pseudoephedrinium [(1*S*,2*S*)-ethyl(2-hydroxy-1-methyl-2-phenylethyl)dimethylammonium] hydroxide was carried out in two steps, first the addition of a methyl group through the *Leucart* reaction, followed by quaternization with ethyl iodide.

In a typical synthesis, 25.00 g of (1*S*,2*S*)-pseudoephedrine are carefully added to a solution of 15.10 g of formaldehyde (37 wt % in aqueous solution) and 23.00 g of formic acid (95 wt %) cooled with an ice bath (careful, exothermic reaction). The mixture is kept under reflux and magnetic stirring overnight. After cooling with an ice bath, the reaction is quenched by adding 18.20 g of hydrochloric acid (37 wt %), and 3 subsequent extractions with diethyl ether are carried out. The aqueous phase is collected, and a 25 wt % NaOH aqueous solution is added until reaching a pH value close to 12. Finally, the organic product is extracted with diethyl ether. The organic phase is collected and dried with potassium carbonate, and the solvent is roto-evaporated, giving an oily product containing (1*S*,2*S*)-N-methyl-pseudoephedrine (80 % yield). ^{1}H NMR ($CDCl_3$): 0.74 (d, 3H), 2.31 (s, 6H), 2.57 (dq, 1H), 4.20 (d, 1H), 5.00 (broad, 1H), 7.22-7.44 (m, 5H). ^{13}C NMR ($CDCl_3$): 7.3, 40.9, 66.9, 75.9, 128.4-129.2, 143.1. [α] = +40º (c = 5.00 g/100mL in methanol).

In the second stage, 20.00 g of (1*S*,2*S*)-N-methyl-pseudoephedrine are dissolved in 400 mL of acetonitrile cooled with an ice bath, to which 34.85 g of iodoethane are dropwise added. The mixture is kept under magnetic stirring at room temperature for 5 days. The solvent is then roto-evaporated, giving a yellowish solid that is exhaustively washed with diethyl ether. The product, (1*S*,2*S*)-N,N-ethyl-methyl-pseudoephedrinium iodide, was obtained with 90 % yield. ^{13}C NMR (D_2O): 7.7, 12.3, 49.1, 49.5, 60.8, 70.9, 74.2, 127.3, 129.1, 140.9. [α] = +40º (c = 5.01 g/100 mL water).

The iodide salt is finally converted into the hydroxide form through ion-exchange with an anionic resin (Amberlite IRN-78, exchange capacity, 4 meq/g; Supelco) ([α] = +66º (c = 2.89 g/100 mL water)). The resulting basic solution is titrated with HCl (0.1 N, Panreac) using phenolphthalein as an indicator, and concentrated by roto-evaporation at 40 ºC up to concentrations of 25-30 wt % of (1*S*,2*S*)-N,N-ethyl-methyl-pseudoephedrinium hydroxide.

The corresponding (1*R*,2*R*)-enantiomer is obtained in the same way, but starting from (1*R*,2*R*)-pseudoephedrine; opposite [α] values were obtained ([α] = –40º for the tertiary amine (c = 5.00 g/100 mL), [α] = –40º for the iodide salt (c = 5.02 g/100 mL), and [α] = –64º for the final hydroxide salt (c = 3.12 g/100 mL)).

B) Details of the synthesis of GTM-3

In a typical preparation, 6.36 g of an aqueous solution of (1*S*,2*S*)-N,N-ethyl-methyl-pseudoephedrinium hydroxide (28.8 wt %) and 0.57 g of GeO_2 are mixed and stirred for 30 minutes, after which 5.76 g of tetraethylorthosilicate (TEOS) are added and stirred until all the ethanol coming up from the hydrolysis of TEOS and the required amount of water to achieve the desired composition are evaporated. 0.34 g of HF (48 %) are then added and manually stirred (with the help of a spatula) until a homogenous thick gel is obtained, giving a gel composition of $0.25EMPS:0.83SiO_2:0.17GeO_2:0.25HF:6.5H_2O$. The gels are introduced into 60 ml Teflon lined stainless steel autoclaves and heated statically at 100 ºC under autogenous pressure for 6 days. The resulting solids were separated by filtration, thoroughly washed with ethanol and water and dried at room temperature overnight. GTM-3 materials could be obtained with Si/Ge ratios of 3 and 5 in the gel. On the other hand, Al-containing GTM-3 catalysts were obtained from gels with (Si+Ge)/Al ratios around 70 with the following molar composition $0.25EMPS:0.007Al_2O_3:0.75SiO_2:0.25GeO_2:0.20HF:6.5H_2O$; Aluminium isopropoxide was added together with TEOS. For these materials, 10% of GTM-3 seeds were added to the synthesis gels to facilitate crystallization.

Calcination of GTM-3 materials was carried out at 550 ºC in air flow for 12 hours; XRD confirmed the resistance of the material to elimination of the organics (Supplementary Fig. S11). After calcination, GTM-3 samples were manipulated under inert atmosphere to avoid contact with ambient humidity.

C) Details of the general physico-chemical characterization

The obtained solids were characterized by powder X-Ray Diffraction (XRD), using a Philips X´PERT diffractometer with CuK_{α} radiation with a Ni filter. Thermogravimetric analyses (TGA) were registered using a Perkin-Elmer TGA7 instrument (heating rate = 20 ºC/min) under air flow. CHN chemical analyses were carried out with a Perkin-Elmer 2400 CHN analyzer. Liquid NMR spectra were recorded with a Bruker Avance III-HD Nanobay 300MHz spectrometer, using a 5mm HBO 1H/X probe. Solid State MAS-NMR spectra of the solid samples were recorded with a Bruker AV 400 WB spectrometer. ^{1}H to ^{13}C CP MAS NMR spectra were recorded using $\pi/2$ rad pulses of 2.75 μs for ^{1}H, a contact time of 3 ms and a recycle delay of 4 s. The spectra were recorded while spinning the samples at *ca* 11.2 kHz. ^{19}F MAS NMR spectra were recorded using $\pi/2$ rad pulses of 2 μ, and a recycle delay of 120 s, while spinning the samples at *ca* 20 kHz. Nitrogen adsorption/desorption isotherms were measured at -196 ºC in a Micromeritics instrument ASAP 2420 device after degassing the sample at 200 ºC for 2 hours.

Field Emission Scanning Electron Microscopy (FE-SEM) was performed using a Philips XL30 S-FEG. Transmission electron microscopy (TEM) images were obtained (after the samples were well ground and dispersed in ethanol) in a JEOL 2000FX microscope operated with an accelerating voltage of 200 kV. Cs-corrected STEM data was collected in a Titan XFEG spherical aberration corrected microscope. This equipment is fitted with a monochromator which facilitates the control of the electron dose and with a CEOS corrector for the electron probe. Because of the low stability and small size of the GTM-3 crystallites, graphene Cu grids were used for image analysis.

D) Details of the polarimetry study

Retention of the enantiopurity of the SDAs during crystallization was analyzed by determining the optical rotation of the SDAs occluded within the zeolite after the hydrothermal treatment. GTM-3 materials prepared with (*S*,*S*)-EMPS (GTM-3/*SS*-EMPS) or (*R*,*R*)-EMPS (GTM-3/*RR*-EMPS) were dissolved in HF and subjected to a polarimetry study. The optical rotation of the SDA solutions (after zeolite dissolution) was determined by using an optical polarimeter (P1000 LED, A. Krüss-Optronic).

GTM-3/SS-EMPS

1.88 g of GTM-3/*SS*-EMPS are suspended in 4.03 g of water, and 3.5 g of HF wt 48 % are carefully added with magnetic stirring until complete dissolution of the zeolite; ^{13}C NMR confirmed the integrity of the released *SS*-EMPS after this dissolution process. 7 g of NaOH aqueous solution (8 M) are carefully added, yielding a solid precipitate. The solids are removed by centrifugation. The supernatant is taken, and water is added until reaching ~11 g of aqueous solution. Further NaOH aqueous solution is then added until reaching a pH of ~13. ^{13}C NMR verified the integrity of EMPS obtained by this protocol (Supplementary Fig. S9). The concentration of EMPS hydroxide is then determined by UV-VIS spectroscopy (after building a calibration curve, and 1:50 dilution to reach the lineal regime) as 2.34 wt %. Finally, the specific rotation of the organic salt (normalized to the concentration) is measured with the polarimeter as +68º, very similar in magnitude and sign to the original value of the pristine *SS*-EMPS hydroxide (+66º), evidencing the retention of enantiopurity of *SS*-EMPS during crystallization of GTM-3/*SS*-EMPS.

GTM-3/RR-EMPS

1.83 g of GTM-3/*RR*-EMPS are suspended in 4.03 g of water, and 3.5 g of HF wt 48 % are carefully added with magnetic stirring until complete dissolution of the zeolite. 8 g of NaOH aqueous solution (8 M) are carefully added, yielding a solid precipitate. The solids are removed by centrifugation. The supernatant is taken, and water is added until reaching ~11 g of aqueous solution. Further NaOH aqueous solution is added until reaching a pH of ~13. The concentration of the organic hydroxide is then determined by UV-VIS spectroscopy as 3.48 wt %. Finally, the specific rotation of the organic salt (normalized to the concentration) is measured with the polarimeter as –58º,

similar in magnitude and sign to the original value of the pristine *RR*-EMPS hydroxide (–64º), evidencing the retention of enantiopurity of *RR*-EMPS during crystallization of GTM-3/*RR*-EMPS.

E) Details of the asymmetric catalytic reaction experiments: ring-aperture of *trans*-stilbene oxide with 1-hexanol

Trans-stilbene oxide (*trans*-2,3-diphenyloxirane) is a chiral reactant and has two enantiomers, (*R*,*R*) and (*S*,*S*). Acid-catalyzed aperture of trans-stilbene oxide with nucleophiles can proceed via two possible mechanisms :[1,2] i) a concerted S_N2-like mechanism where protonation of O and attack by the nucleophile (1-hexanol) from the opposite side occurs in a concerted way, giving place to (*R*,*S*) and (*S*,*R*)-*u*-products (with inversion of configuration) from (*R*,*R*) and (*S*,*S*)-reactants, respectively, or ii) a S_N1-like mechanim where the epoxide is first protonated, giving place to the corresponding carbocation with a C planar geometry where attack from the nucleophile can occur from both sides, giving place to a mixture of *u*- [(*R*,*S*) and (*S*,*R*), with inversion of configuration] or *l*- [(*R*,*R*) and (*S*,*S*), with retention of configuration] products. *U*-species are usually the most abundant products with homogeneous acid catalysts.

Catalytic experiments were performed with 20 wt % of catalytst, and 10 mg/ml of racemic *trans*-stilbene oxide dissolution in 1-hexanol; catalytic reaction was carried out at 30 ºC, and aliquots were extracted at different time intervals. Calcined GTM-3 samples were manipulated under inert atmosphere. Evolution of the reaction was followed by HPLC with chiral stationary phases; (Supplementary Fig. S17).

The evolution of the reactants, inversion (*u*) and retention (*l*) products was followed by HPLC (Agilent 1200 Infinity Series, with Quad Pump and DAD detector) using chiral chromatographic columns Chiralpak IG and Chiralcel OD-H (Daicel); purity of the peaks was analyzed by UV-VIS spectroscopy. Reactants (*RR* and *SS*) and retention (*l*, *RR* and *SS*) products were analyzed with Chiralcel OD-H column, using mobile phases of 97(hexane):3(isopropanol) (flow 1.2 ml/min) at 0 ºC for reactants (λ = 235 nm), and 93(hexane):7(isopropanol) (flow 1.2 ml/min) at 25 ºC for retention products (λ = 215 nm). Inversion (*u*, *RS* and *SR*) products were analyzed with Chiralpac IG column, using a mobile phase of 75(hexane):25(isopropanol) (flow 1.2 ml/min) at 0 ºC (λ = 215 nm) (Supplementary Fig. S17).

Standards for the *u*- and *l*-products were prepared from the reaction of 1-hexanol with *trans*-stilbene oxide or *cis*-stilbene oxide, respectively, catalyzed by homogeneous sulfuric acid; *u*/*l* products were characterized by liquid NMR (Supplementary Fig. S16). These standards were used to identify the corresponding peaks in the HPLC analyses.

F) Details of the characterization of acid sites by FTIR spectroscopy analysis of adsorbed pyridine

Fourier transform infrared (FTIR) spectra were recorded with a Thermo Nicolet Nexus 670 FTIR spectrophotometer, equipped with an MCT cryodetector. The as-made sample SS-GTM-3 was pressed into a self-supporting wafer (thickness ca. 10 mg/cm^2), calcined in synthetic air at 550°C for 12 h, transferred under dry nitrogen atmosphere to a transmission IR cell made of glass and provided with CaF_2 windows, and degassed in vacuum (ca. 10^{-3} mbar) at 450°C for 12 h. The pre-treated sample wafer was kept in contact with excess pyridine vapor at room temperature for 10 min and then evacuated for 30 min at increasing temperatures from 50 to 300°C. FTIR spectra were acquired in the mid-IR range at 4 cm^{-1} resolution by averaging 250 scans. The spectra of adsorbed pyridine were computed by subtracting the spectrum of the calcined sample from the spectra collected after pyridine adsorption and thermodesorption. Density of acid sites was calculated from the integral intensity of the band at 1543 cm^{-1} (Brønsted sites) and at 1452 and 1458 cm^{-1} (Lewis sites) using the extinction coefficients determined by Emeis.[3] Overlapping bands were deconvoluted into Gaussian components using Microcal OriginPro v9.8 software.

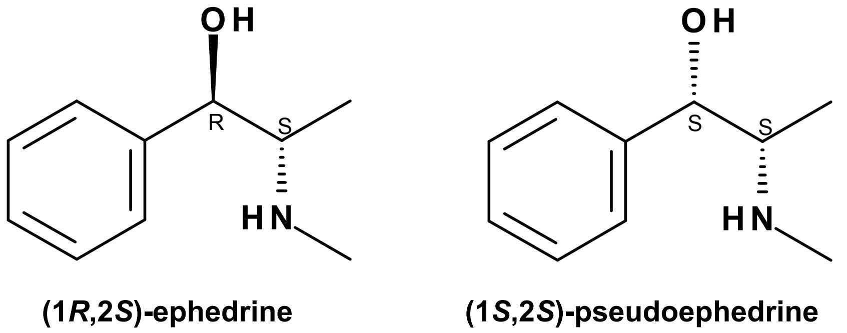

Supplementary Fig. S1. Molecular structure of diastereomeric alkaloids (1*R*,2*S*)-ephedrine (left) and (1*S*,2*S*)-pseudoephedrine (right) available from the chiral pool.

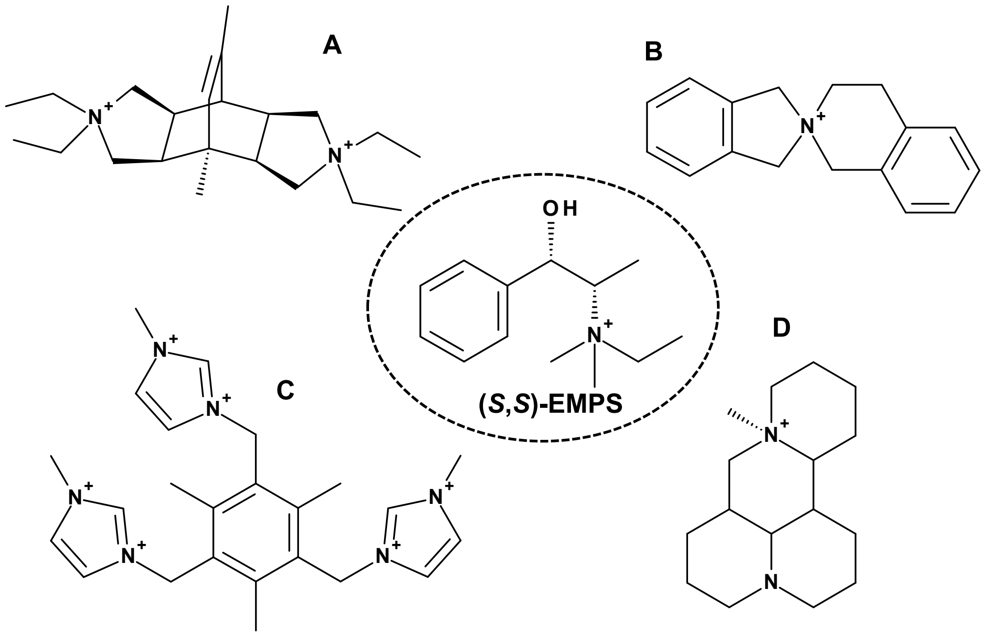

Supplementary Fig. S2. SDAs that have been reported to direct the formation of the –ITV framework, compared with (*S*,*S*)-EMPS.

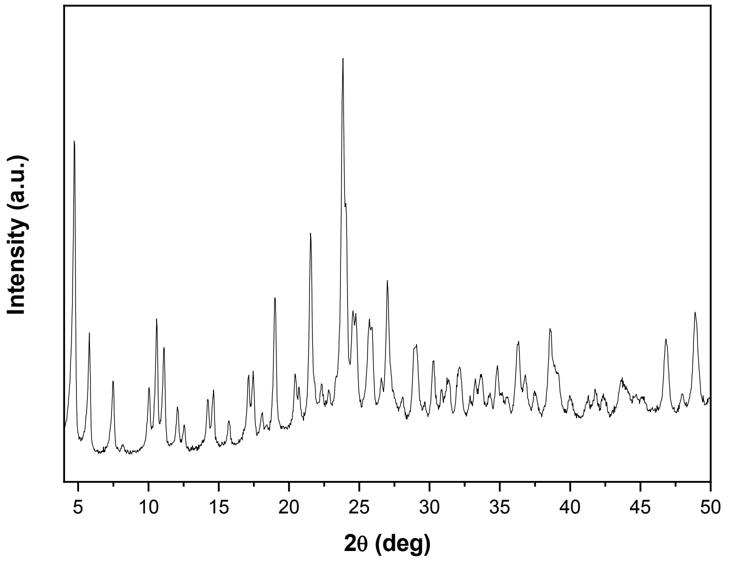

Supplementary Fig. S3. XRD pattern of as-made GTM-3.

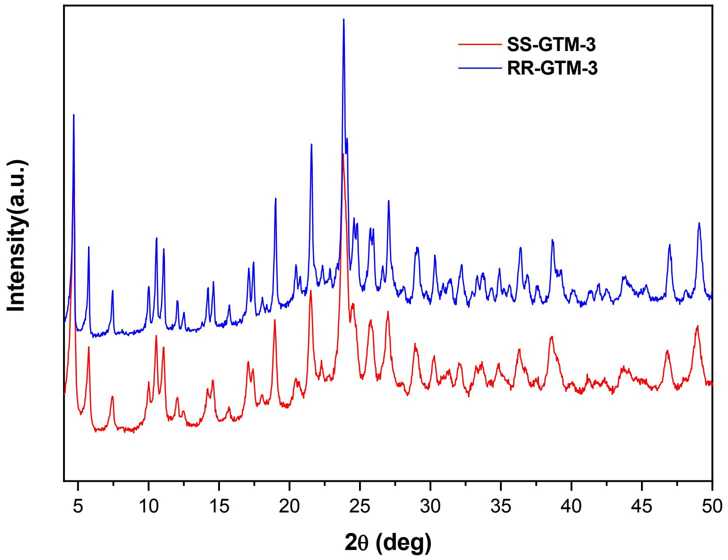

Supplementary Fig. S4. XRD patterns of as-made GTM-3 prepared with (1*R*,2*R*)- (top) or (1*S*,2*S*)- (bottom) enantiomers of EMPS.

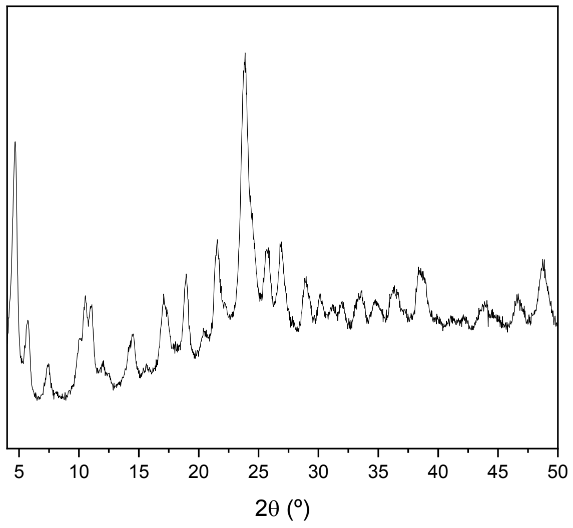

Supplementary Fig. S5. XRD pattern of as-made GTM-3 prepared at 60 ºC for 14 days.

Although ITV was the only crystalline phase observed, XRD patterns of GTM-3 showed the presence of some remaining amorphous material in the samples, which increased upon reduction of the crystallization temperature (compare Figure S5 with Figure S3 and S4). Such unreacted material might be a consequence of the low crystallization temperature required to avoid the thermal decomposition of the EMPS cations (below 110 ºC), which are relatively unstable under hydrothermal conditions.

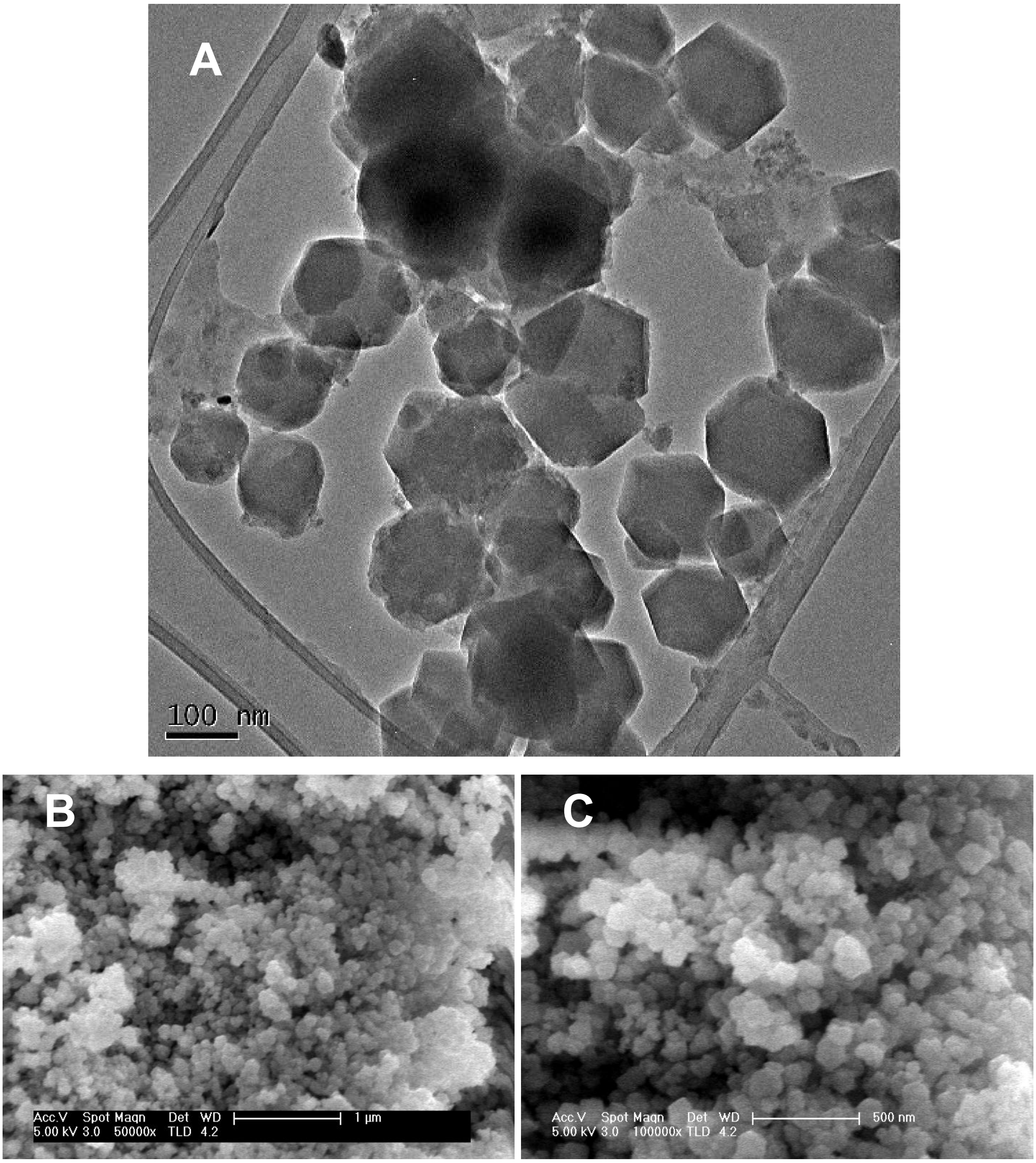

Supplementary Fig. S6. A. TEM picture of as-made GTM-3. **B,C:** FESEM pictures of as-made GTM-3 showed cubic-like crystals of nanometer size, as commonly observed for this structure.

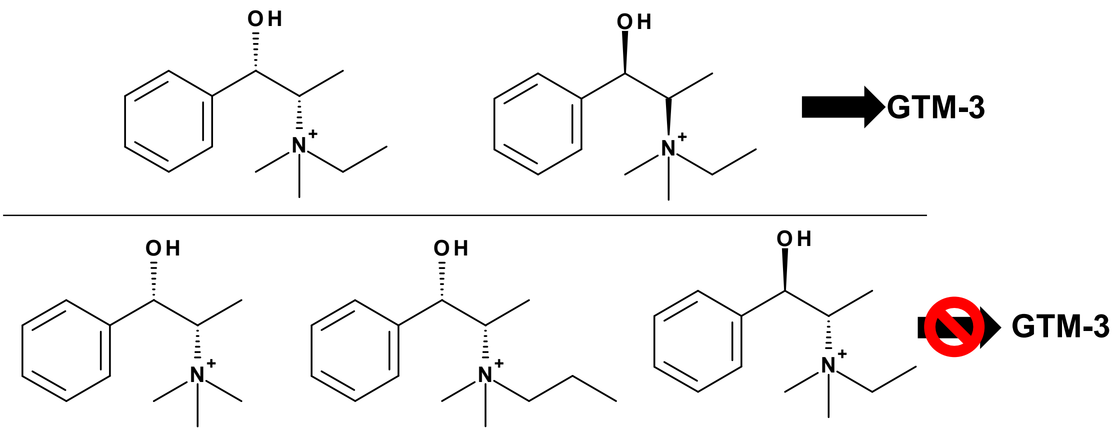

Supplementary Fig. S7. SDAs that directed the formation of the –ITV framework (top, *SS*-EMPS, left, and *RR*-EMPS, right), and related cations that did not (bottom). Only amorphous materials with traces of BEC (polymorph C of zeolite beta) were observed in the solid products when using these alternative cations.

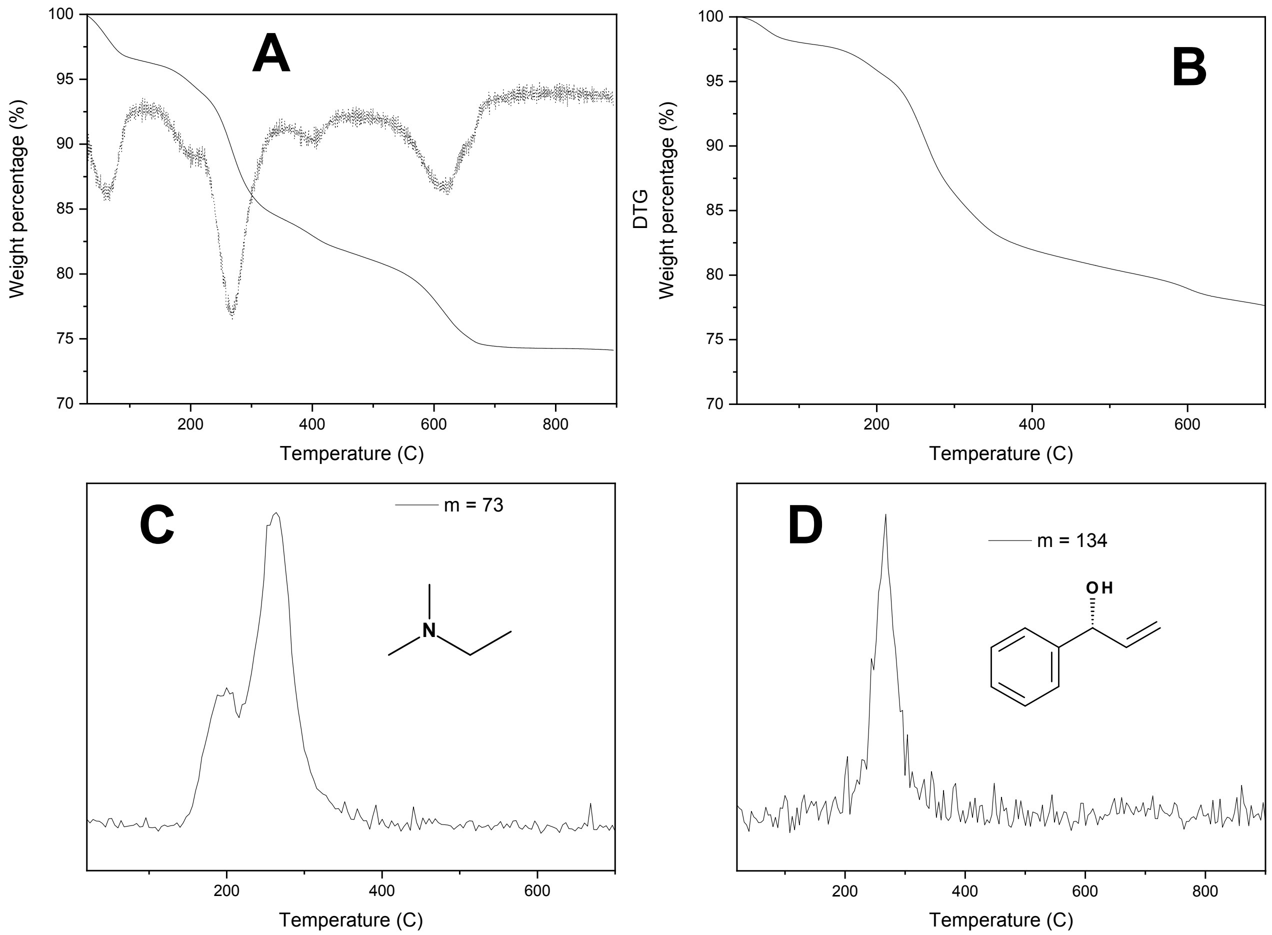

Supplementary Fig. S8. A. TGA of GTM-3 in air (solid line), and weight derivative (right, DTG). **B.** TGA of GTM-3 in He (**B**), coupled to mass spectrometry (**C** and **D**), showing the release of the decomposition products of EMPS.

Supplementary Tab. S1. CHN elemental analysis of GTM-3 (two analyses of the same sample are shown); *theoretical C/N ratios are shown between brackets.

	% C	% H	% N	C/N*
**GTM-3_1**	14.29	2.68	1.31	12.7 (13)
**GTM-3_2**	14.28	2.63	1.29	12.8 (13)

Thermogravimetric (Figure S8-A) and CHN elemental analysis (Table S1) of GTM-3 showed a very large organic content of 19.3 %, corresponding to a packing value of 18 EMPS per ITV unit cell; such amount of organic cations is very close to the number of D4R units in the ITV framework (20), and therefore of the negative charges provided by F^- anions occluded within those D4R units. TGA (in He) coupled to mass spectrometry (Figure S8-B,C,D) showed the release of the expected decomposition products of EMPS upon Hofmann elimination. CHN analysis showed a C/N ratio of 12.8 (Table S1), very close to the theoretical value of 13 in EMPS.

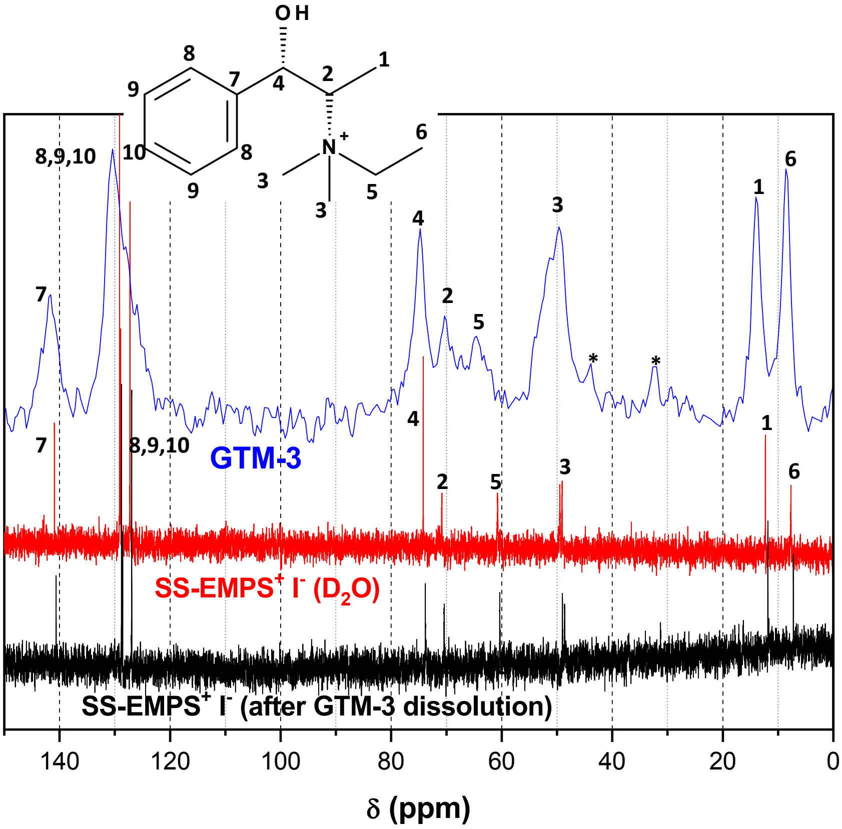

Supplementary Fig. S9. ^{13}C NMR spectra of EMPS iodide in aqueous solution (D_2O, red), confined within GTM-3 (top, blue), and in aqueous solution after dissolution of GTM-3 (black); * indicates spinning side bands.

All the resonances corresponding to the different C atoms of the pristine EMPS iodide salt (red line, middle) are present in GTM-3 (blue line, top), evidencing the integral incorporation of EMPS. However, the band corresponding to C5 is shifted to lower fields (from 61 in aqueous solution to 65 ppm in GTM-3); in order to ensure that this shift is a consequence of confinement in GTM-3 and not a degradation of the ethyl group, a sample of GTM-3 zeolite was dissolved in aqueous HF, and the ^{13}C NMR spectrum of the resulting solution (black line, bottom) again showed exactly the same bands as the original EMPS iodide solution. This clearly evidences that such shift of C5 is a consequence of confinement in the zeolite.

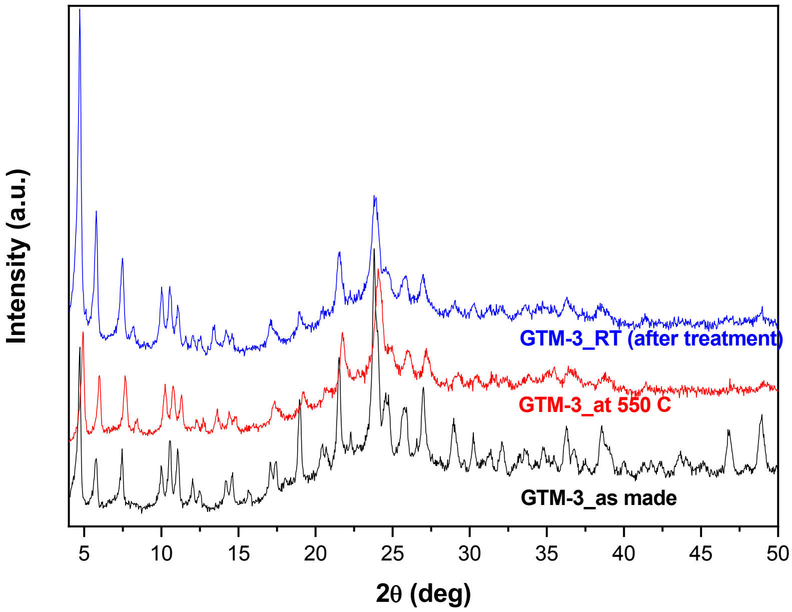

Supplementary Fig. S10. In-situ XRD patterns of GTM-3 at increasing temperatures: bottom: original GTM-3 (black line); middle: GTM-3 at 550 ºC (red line); top: GTM-3 after thermal treatment up to 550 ºC and subsequently cooled at room temperature (blue line).

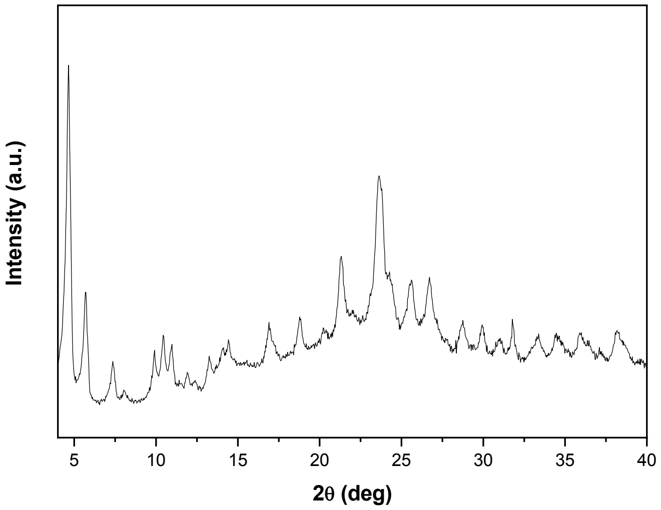

Figure S11. XRD pattern (performed under inert atmosphere) of calcined GTM-3.

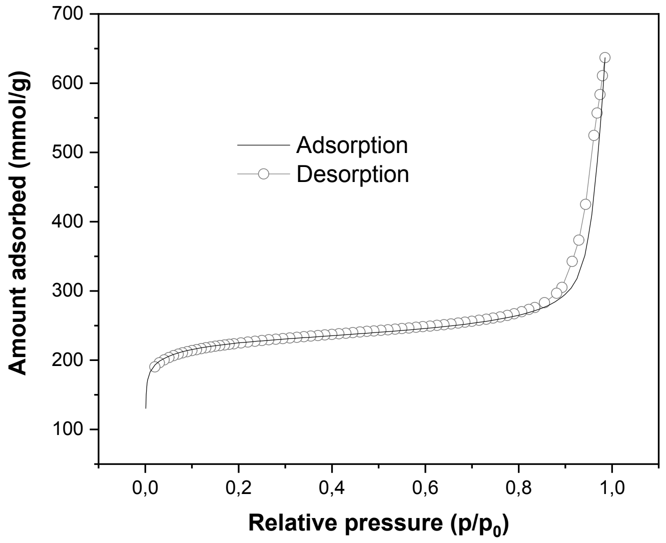

Supplementary Fig. S12. N_2 adsorption/desorption isotherm of calcined GTM-3.

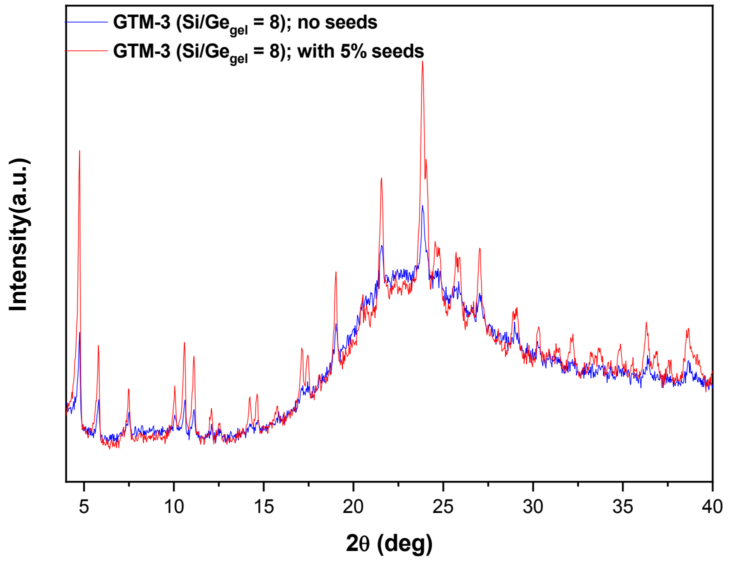

Figure S13. XRD patterns of GTM-3 materials obtained with a Si/Ge ratio of 8 in the gel at 100 ºC for 14 days, in the absence (blue line) or in the presence (red line) of seeds (5 wt % with respect to SiO_2+GeO_2).

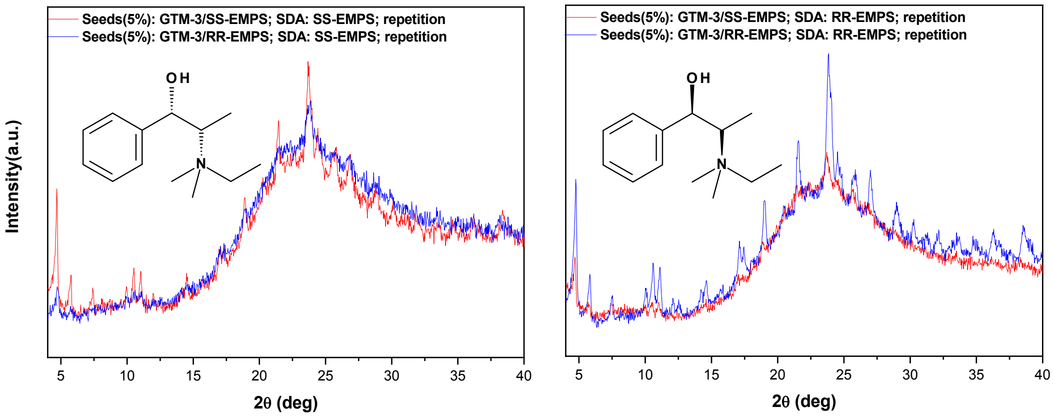

Figure S14. Repetition of enantioselective crystallization experiments: XRD patterns of samples prepared with *SS*-EMPS (left) or *RR*-EMPS (right) as SDA in the gel, using 5 % of GTM-3 seeds previously prepared with *SS*-EMPS (red lines) or *RR*-EMPS (blue lines). Crystallization was carried out at 100 ºC for 72 hours.

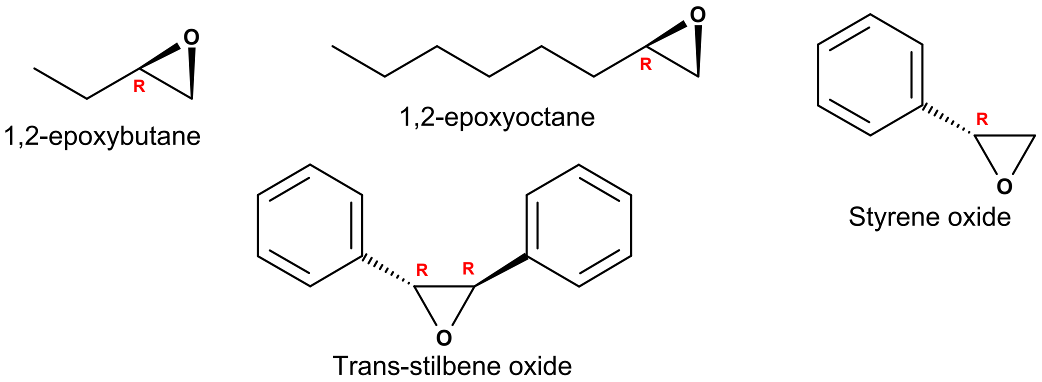

Supplementary Fig. S15. Molecular simulations for the selection of the epoxide: Molecular structure of different chiral epoxides studied; only *R* (or *R*,*R*) enantiomers are shown.

Table S2. Molecular dimensions (expressed as averaged molecular shadow lengths during MD simulations) of the different epoxides studied computationally.

Molecular shadow lengths	1,2-epoxybutane	1,2-epoxyoctane	Styrene oxide	Trans-stilbene oxide
X	7.6	11.5	9.5	13.5
Y	5.3	6.4	6.8	6.9

Table S3. Averaged interaction energies (in kcal/mol epoxide) of one enantiomer of the epoxide molecule loaded in the two enantiomorphic -ITV frameworks; enantiodifferentiation energies (ΔE) are expressed as the difference between the interaction energy of the *R* enantiomer minus that of the *S* enantiomer for a given -ITV polymorph.

**Epoxide**	$P4_132$			$P4_332$		
	R (RR)	S (SS)	ΔE(R-S)	R (RR)	S (SS)	ΔE(R-S)
**1,2-epoxybutane**	-16.18	-16.23	+0.05	-16.19	-16.15	-0.04
**1,2-epoxyoctane**	-25.15	-25.11	+0.04	-25.12	-25.07	-0.05
**Styrene oxide**	-22.85	-22.44	-0.41	-22.47	-22.91	+0.44
**Trans-stilbene oxide**	-34.21	-33.49	**-0.72**	-33.59	-34.25	**+0.66**

Molecular simulations based on the Dreiding force-field[4] were used in order to guide our selection of a proper chiral epoxide substrate whose chirality is able to be recognized by the enantiomorphic polymorphs of -ITV ($P4_132$ and $P4_332$). Four chiral epoxides with different size (including aliphatic and aromatic chains) were studied as potential reactants: 1,2-epoxybutane, 1,2-epoxyoctane, styrene oxide and *trans*-stiblene oxide (**A**). Dimensions of the epoxides are expressed as the three molecular shadow lenghts averaged along 100 ps NVT MD simulations (**B**). The chiral-recoginition ability of the -ITV framework for the chiral epoxides was estimated by loading one epoxide molecule (each enantiomer separately) on each of the two enantiomorphic ITV polymorphs ($P4_132$ and $P4_332$) and running 1000 cycles of simulated annealing calculations (only hydroxyl groups of the ITV framework and the epoxide molecules were allowed to relax). The interaction energies were calculated by subtracting to the final energy the energy of both the ITV framework and the isolated epoxide molecule, and were averaged along the 1000 annealing cycles (**C**).

The condition for ITV to recognize the chirality of the epoxide molecule involves the enantiodifferentiation energies of the two polymorphs to be similar in magnitude but opposite in sign; the higher the energy, the higher the enantio-discrimination ability. For our set of epoxides, the aliphatic 1,2-epoxybutane and 1,2-epoxyoctane did not satisfy such enantio-discrimination condition (the enantiodifferentiation energy were very close to 0), probably because of their size (compared to that of the ITV channels) and the high conformational flexibility. In contrast, both aromatic epoxides, styrene and *trans*-stilbene oxides, satisfied such condition, and a notably higher enantio-differentiation energy was observed for the latter (~0.7 kcal/mol), probably because of its larger size (**B**) and the higher rigidity provided by the presence of two aromatic rings. Thus, we selected *trans*-stilbene oxide as substrate for the catalytic asymmetric reaction.

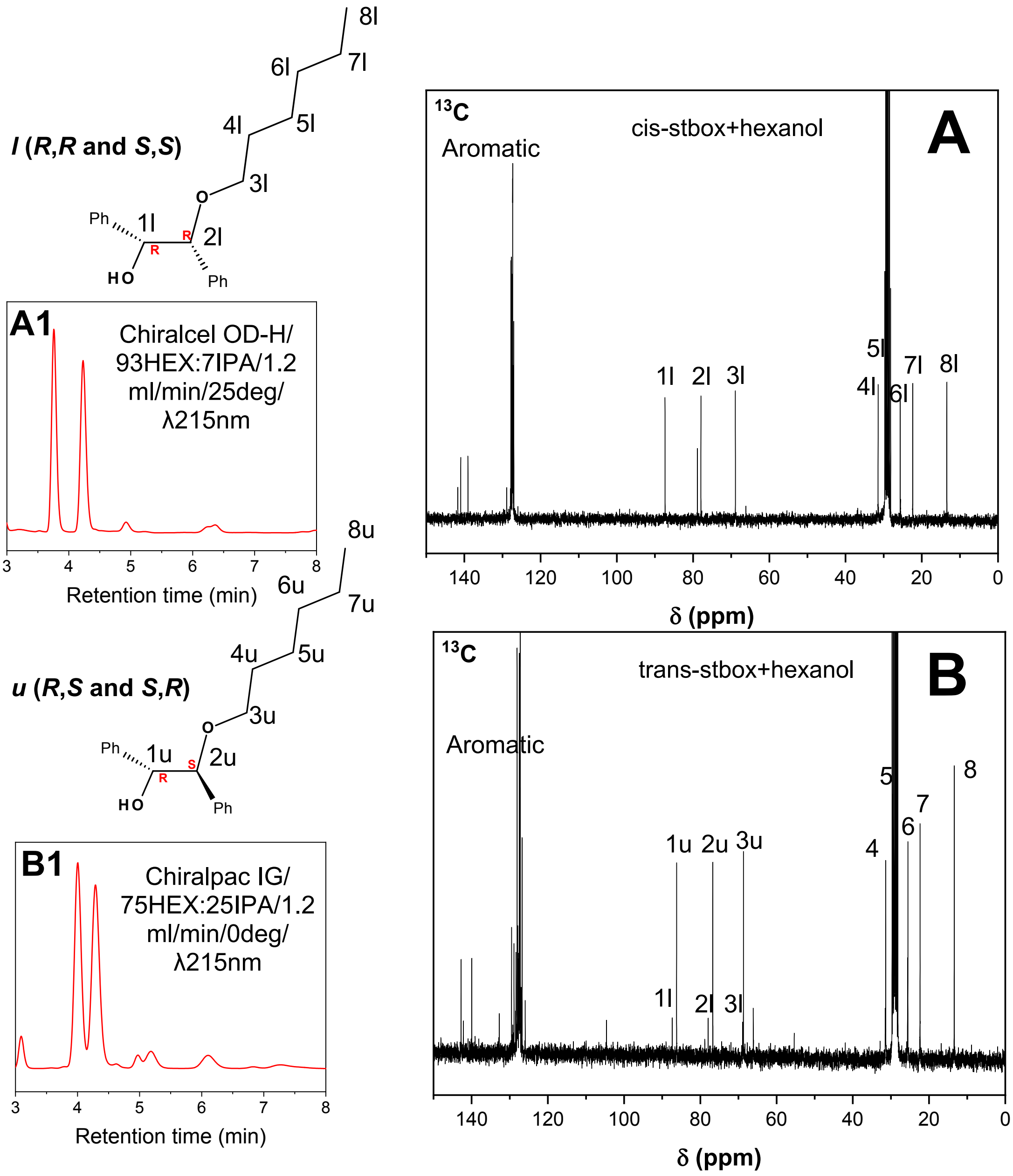

Supplementary Fig. S16. NMR characterization of the stilbene oxide aperture with 1-hexanol. Standards of the products of the *trans*-stilbene oxide ring aperture with 1-hexanol were prepared using sulfuric acid as catalyst. **A.** ^{13}C NMR of products of the *cis*-stilbene oxide ring aperture with 1-hexanol catalyzed by sulfuric acid, where the products have *l*-configuration (*R*,*R* and *S*,*S*), and the corresponding HPLC chromatogram in the conditions used for its determination (**A1**). **B.** ^{13}C NMR of products of the *trans*-stilbene oxide ring aperture with 1-hexanol catalyzed by sulfuric acid, where the main products have *u*-configuration (*R*,*S* and *S*,*R*), although in this case a minor amount of *l*-products are also observed (*u*/*l* ratio ~ 9), and the corresponding HPLC chromatogram in the conditions used for its determination (**B1**).

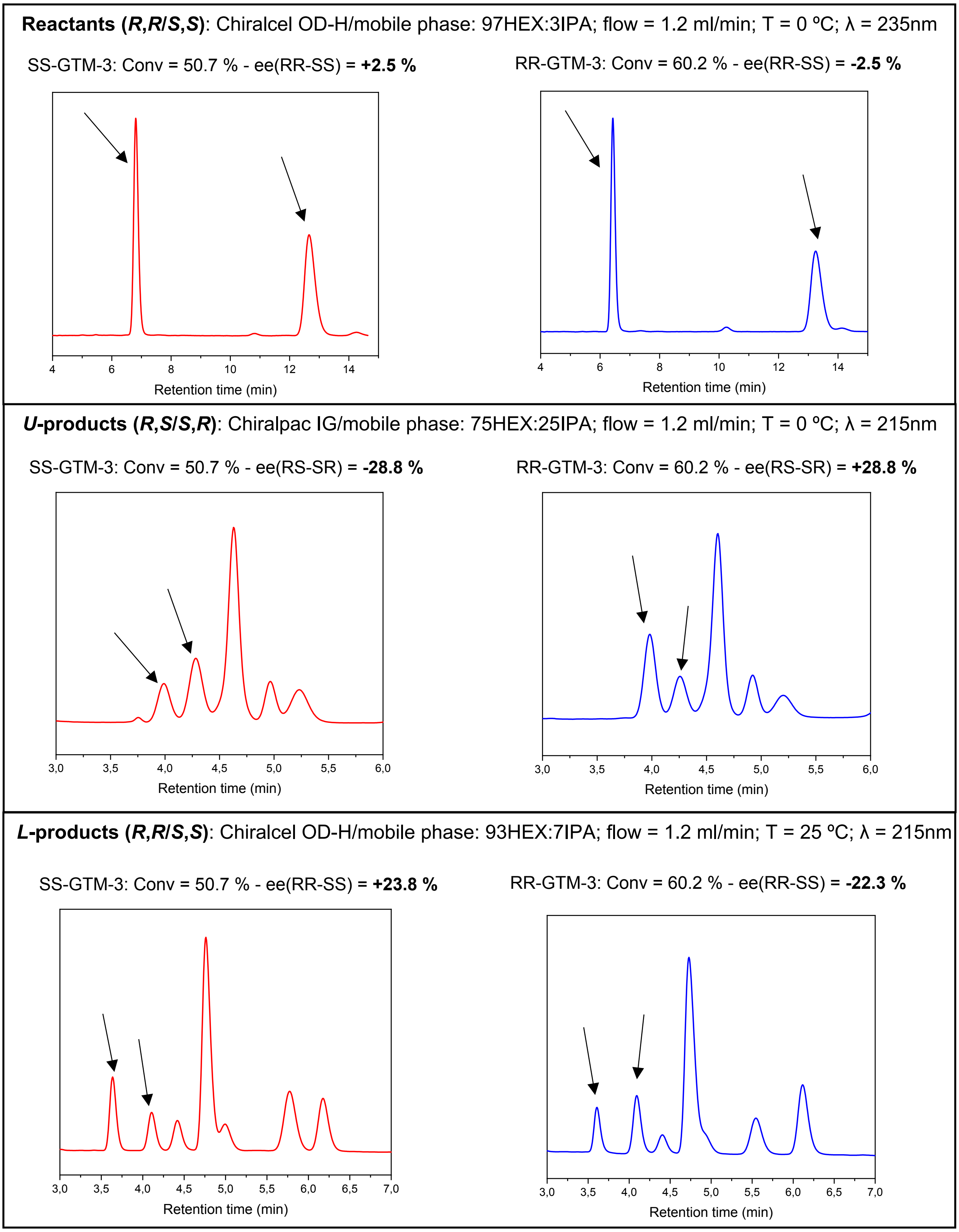

Supplementary Fig. S17. HPLC settings and selected chromatograms for the determination of the enantiomeric excesses of reactants (top), *u*-products (middle) and *l*-products (bottom) for the *trans*-stilbene oxide ring-aperture with 1-hexanol.

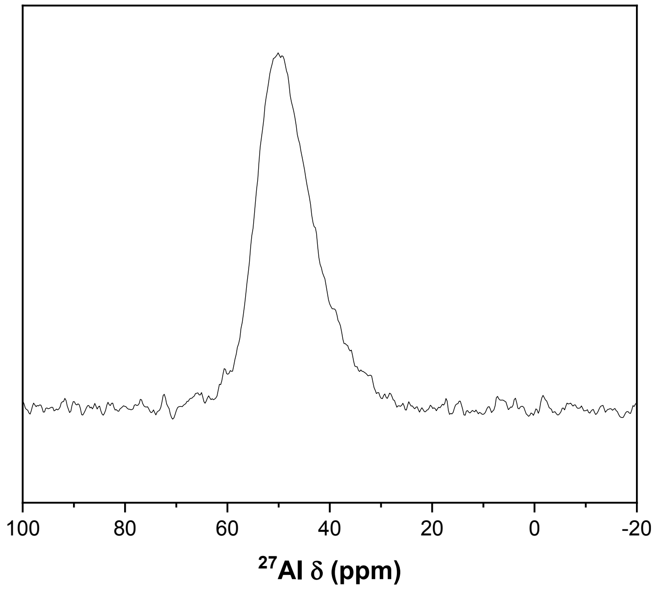

Supplementary Fig. S18. ^{27}Al MAS NMR of Al-containing GTM-3 (prepared with T/Al ratio of 70 in the gel). The band at 50 ppm is characteristic of Al in tetrahedral zeolite framework positions.

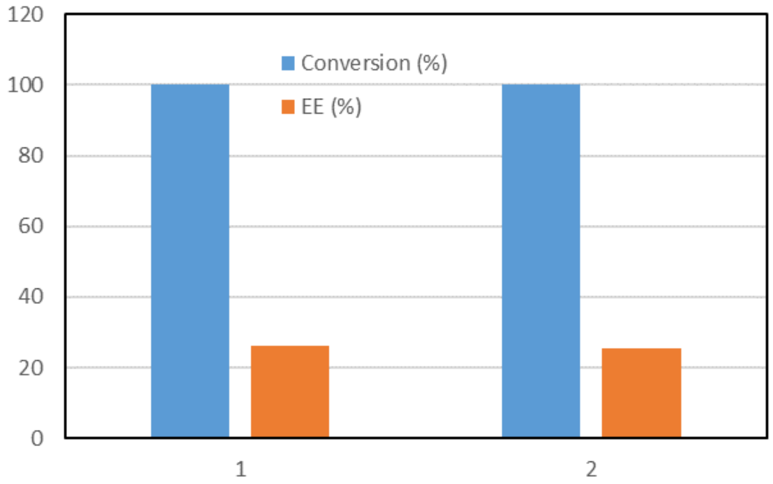

Supplementary Fig. S19. Recycle of GTM-3 catalyst.

GTM-3 catalyst could be recycled, maintaining the same conversion and *ee*. Deactivation of GTM-3 upon this type of catalysis is expected to be low because of the extra-large pores, and the moderate conditions at which the reaction takes place (30 ºC).

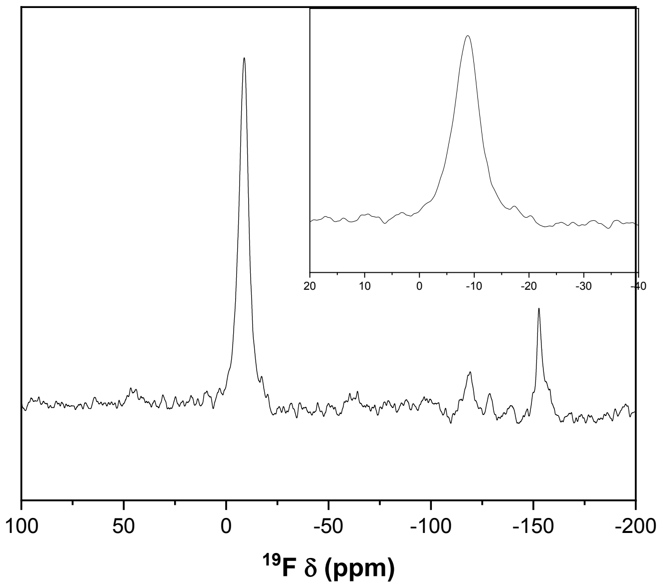

Supplementary Fig. S20. ^{19}F MAS NMR of GTM-3.

Following the assignment proposed by Camblor et al. for the STW framework,[5] the main band at -9 ppm is characteristic of F^- in D4Rs with several Ge atoms, and the broadness of the band possibly reflects a mixture of F^- in D4Rs with Ge pairs (band at -7.5 ppm) and with Ge close clusters (band at -10.5 ppm). A very small shoulder at -17 ppm might indicate the presence of a very minor, if at all, amount of isolated Ge in D4Rs. The additional bands at around -120 ppm are assigned to $[SiF_x(OH)_{6-x}]^{2-}$ impurity amorphous species or to surface fluorosilicate species,[6] while the band at -153 ppm has been previously assigned to dissociative F^- in the channels.[7]

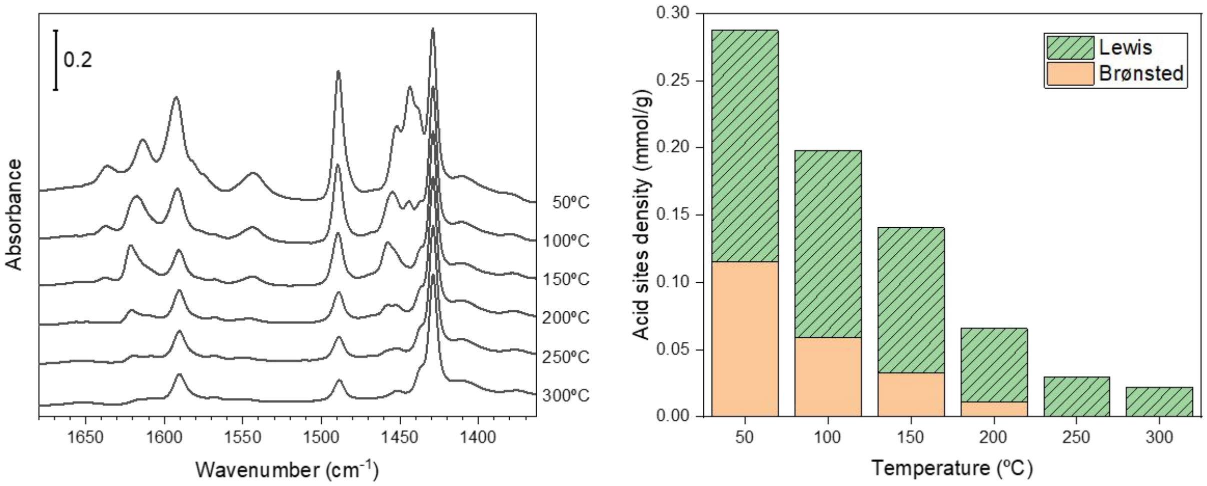

Supplementary Figure S21. FTIR spectra recorded after adsorption of pyridine at room temperature on the calcined sample SS-GTM-3 and subsequent evacuation at increasing temperature (left) and estimated amount of pyridine adsorbed on acid sites (right).

Characterization of acid sites by FTIR spectroscopy analysis of adsorbed pyridine.

The spectra show bands characteristic of pyridine coordinatively bonded to Lewis acid sites at 1613, 1489 and 1452 cm^{-1} (which evolve into two additional components at 1621 and 1458 cm^{-1} after evacuation at 100 °C), pyridinium ions bonded to Brønsted sites at 1637, 1543 and 1489 cm^{-1} and H-bonded pyridine at 1593, 1489 and 1444 cm^{-1}.[8] Bands corresponding to H-bonded pyridine and pyridinium ions are fully removed after evacuation at 100 and 200 °C, respectively, whereas those corresponding to pyridine coordinatively bonded to Lewis sites are still present after evacuation at 300 °C although strongly decrease at temperatures above 150 °C. Furthermore, additional bands are found at 1591, 1489, 1438 (shoulder) and 1429 cm^{-1} which retain most of their initial intensity even after evacuation at 300 °C. This later group of bands that, to the best of our knowledge, have not been previously reported for other germano-silicates, should correspond to pyridine more strongly bonded to other acid sites that seem to be characteristic of this material.

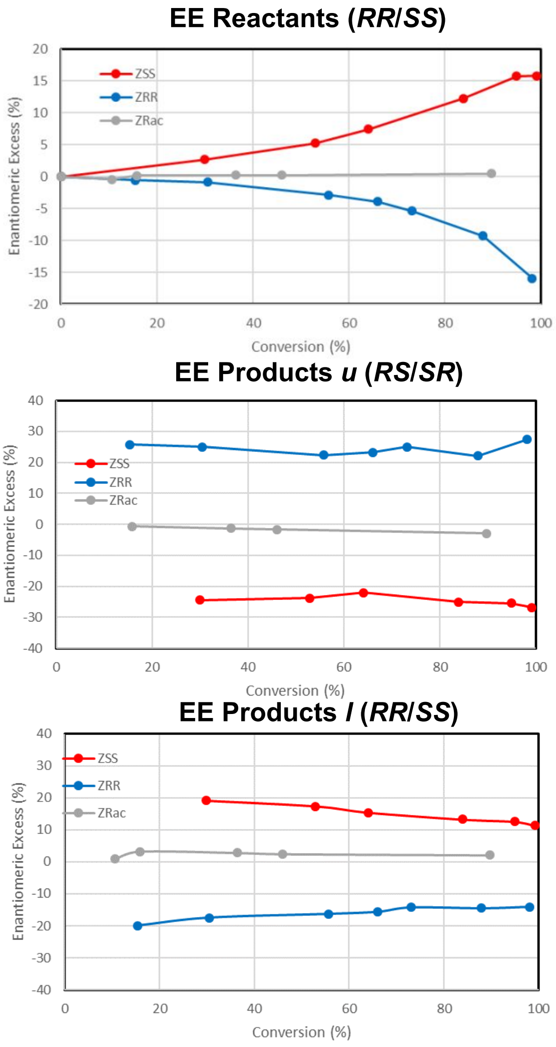

Supplementary Fig. S22. Enantiomeric excesses (%) of reactants (top), *u*-products (middle) and *l*-products (bottom) as a function of conversion, using Al-containing GTM-3 catalysts obtained with *SS*-EMPS (red), *RR*-EMPS (blue) or an artificial racemic mixture of both (grey), for the *trans*-stilbene epoxide aperture with 1-hexanol.

Minor deviations from *ee* = 0 expected for racemic-GTM-3 are due to the fact that the racemic EMPS was artificially prepared from the enantiomerically-pure hydroxide EMPS solutions, and minor errors coming from the determination of the concentration might involve a slight enrichment in one of the enantiomers.

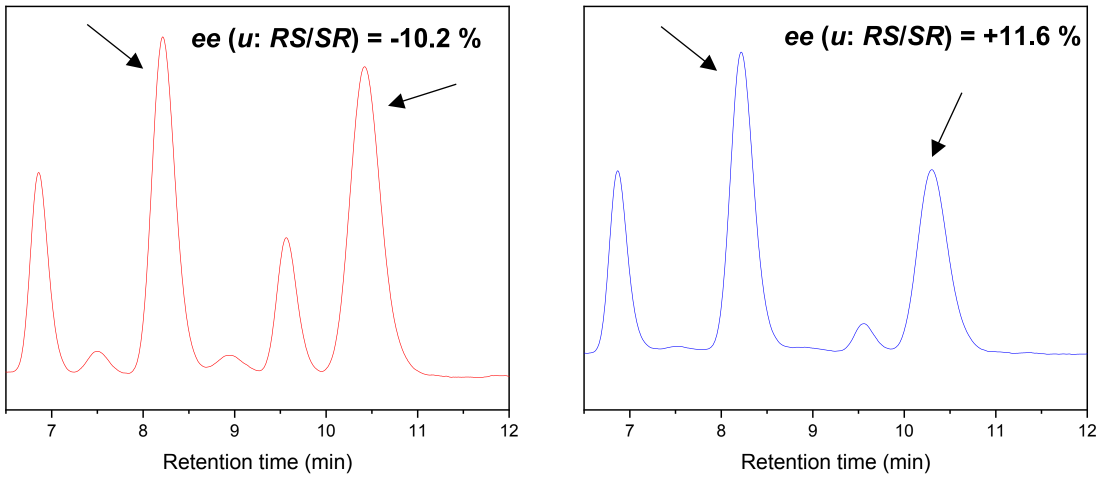

Supplementary Fig. S23. HPLC chromatograms (and enantiomeric excesses (%)) of *u*-products for the *trans*-stilbene epoxide aperture with ethanol. HPLC conditions: Chiralcel OD-H/mobile phase: 95HEX:5IPA; flow = 1.2 ml/min; T = 2 ºC; λ = 215nm.

References

[1] Masashi, I.; Yoshio, T.; Toshio, S.; Katsuhiko, I. Medium effects on Stereochemistry of acid-catalyzed ethanolysis of trans-2,3-diphenyloxirane, *Bull. Chem. Soc. Jap.* **1978**, *51*, 2098-2107.

[2] Parker, R. E.; Isaacs, N. S. Mechanisms of epoxide reactions, *Chem. Rev.* **1959**, *59*, 737-799.

[3] Emeis, C.A., Determination of integrated molar extinction coefficients for infrared absorption bands of pyridine adsorbed on solid acid catalysts, *J. Catal.* **1993**, *141*, 347-354.

[4] Mayo, S. L.; Olafson, B. D.; Goddard, W. A. DREIDING: a generic force field for molecular simulations, *J. Phys. Chem.* **1990**, *94*, 8897-8909.

[5] Rigo, R. T.; Balestra, S. R. G.; Hamad, S.; Bueno-Pérez, R.; Ruiz-Salvador, A. R.; Calero, S.; Camblor, M. A. The Si–Ge substitutional series in the chiral STW zeolite structure type. *J. Mater. Chem. A* **2018**, *6*, 15110–15122.

[6] Lu, P.; Gómez-Hortigüela, L.; Camblor, M. A. Synthesis of Pure Silica MWW Zeolite in Fluoride Medium by Using an Imidazolium-Based Long Dication. *Chem. Eur. J.* **2019**, *25*, 1561-1572.

[7] Hua, W.; Chen, H.; Yu, Z.-B.; Zou, X.; Lin, J.; Sun, J. A Germanosilicate Structure with 11x11x12-Ring Channels Solved by Electron Crystallography. *Angew. Chem. Int. Ed.* **2014**, *53*, 5868-5871.

[8] Podolean, I.; Zhang, J.; Shamzhy, M.; Pârvulescu, V. I.; Čejka, J. Solvent-free ketalization of polyols over germanosilicate zeolites: the role of the nature and strength of acid sites. *Catal. Sci. Technol.* **2020**, *10*, 8254–8264.
